# Mannose and PMI depletion overcomes radiation resistance in HPV-negative head and neck cancer

**DOI:** 10.1186/s12964-025-02204-0

**Published:** 2025-04-21

**Authors:** Tongchuan Wang, Connor Brown, Niamh Doherty, Niall M. Byrne, Rayhanul Islam, Meabh Doherty, Jie Feng, Cancan Yin, Sarah Chambers, Lydia McQuoid, Letitia Mohamed-Smith, Karl T. Butterworth, Emma M. Kerr, Jonathan A. Coulter

**Affiliations:** 1 School of Pharmacy, Queens University Belfast, 97 Lisburn Road, Belfast, BT9 7BL UK; 2 Patrick G. Johnston Centre for Cancer Research, Queens University Belfast, 97 Lisburn Road, Belfast, BT9 7BL UK

**Keywords:** Mannose, Tumour metabolism, Head and neck cancer, Phosphomannose isomerase, Radiotherapy

## Abstract

Radiotherapy is critical component of multidisciplinary cancer care, used as a primary and adjuvant treatment for patients with head and neck squamous cell carcinoma. This study investigates how mannose, a naturally occurring monosaccharide, combined with phosphomannose isomerase (PMI) depletion, enhances the sensitivity of HPV-negative head and neck tumour models to radiation. Isogenic PMI knockout models were generated by CRISPR/Cas9 gene editing, yielding a 20-fold increase in sensitivity to mannose in vitro, and causing significant tumour growth delay in vivo. This effect is driven by metabolic reprogramming, resulting in potent glycolytic suppression coupled with consistent depletion of ATP and glycolytic intermediates in PMI-depleted models. Functionally, these changes impede DNA damage repair following radiation, resulting in a significant increase in radiation sensitivity. Mannose and PMI ablation supressed both oxygen consumption rate and extracellular acidification, pushing cells towards a state of metabolic quiescence, effects contributing to increased radiation sensitivity under both normoxic and hypoxic conditions. In 3D-tumoursphere models, metabolic suppression by mannose and PMI depletion was shown to elevate intra-tumoursphere oxygen levels, contributing to significant in vitro oxygen-mediated radiosensitisation. These findings position PMI as a promising anti-tumour target, highlighting the potential of mannose as a metabolic radiosensitiser enhancing cancer treatment efficacy.

**Supplementary Information:**

The online version contains supplementary material available at 10.1186/s12964-025-02204-0.

## Abstract (plain language summary)

Radiotherapy is a vital treatment for head and neck cancer, however, many of these tumours poses areas that are poorly oxygenated, a feature that that causes resistance to radiotherapy. This study explores how mannose, a natural sugar, combined with reducing the activity of a protein called phosphomannose isomerase (PMI), makes HPV-negative head and neck tumours more sensitive to the effects of radiation. By dampening the ability of PMI to metabolise mannose, we report that the accumulation mannose metabolites disrupt tumour metabolism, depleting the tumour cell supply of energy (ATP), supressing the ability to repair DNA damage following radiation, and importantly helping to overcome resistance to radiation treatment caused by low-oxygen conditions. In 3D-tumour models, this combination resulted in increased tumoursphere oxygenation, further enhancing the effectiveness of radiation. These findings suggest PMI coupled with mannose as a promising target to improve cancer treatment outcomes.

**Supplementary Information:**

The online version contains supplementary material available at 10.1186/s12964-025-02204-0.

## Introduction

Head and neck squamous cell carcinoma (HNSCC) refers to tumours arising within the oral cavity, larynx, pharynx, hypopharynx, nasal cavity, and salivary glands [1]. It includes both human papillomavirus (HPV) positive (HPV+) and HPV negative (HPV−) cancers. HPV + HNSCC, primarily found in the oropharynx, is linked to a viral aetiology, while HPV − HNSCC, associated with smoking and alcohol consumption, occur sporadically across the oral cavity, often in areas less suited to surgical resection [2, 3]. Consequently, HPV + HNSCC has a better prognosis than HPV − cancer, with standard treatments like surgery and radiotherapy (RT) contributing to improved outcomes [3, 4].

Treatment for advanced locoregional HNSCC typically involves surgery followed by adjuvant radiation therapy (aRT). Radiation doses are tailored to tumour stage: low-risk (Stage I/II) cases receive 45–50 Gy, high-risk regions 56–60 Gy, and gross disease up to 70 Gy, delivered in 1.8–2 Gy fractions over 6–7 weeks [1, 5]. HPV + tumours respond better to RT and concurrent chemotherapy (cisplatin), significantly improving overall survival (HR 0.569, CI 0.371–0.872, *p* = 0.01) [2]. In contrast, RT alone provides limited benefit for HPV − patients, with total doses constrained by treatment-related adverse effects [2].

The ARTFORCE trial (NCT01504815) utilised ^18^F-fluorodeoxyglucose positron emission tomography (^18^F-FDG-PET) to target radioresistant tumour sub-volumes with dose escalation (median 77 Gy, max 84 Gy) based on regions with the highest standardised uptake values (SUVmax) [6]. Stratification by nodal stage (N0-1) revealed significant improvements in locoregional control for both HPV + and HPV − tumours (HR 0.21, CI 0.05–0.93), however, no overall survival benefit was observed [7]. Possible explanations include insufficient dose escalation, with less than 2% of the boosted region receiving the maximum dose, and limitations of FDG-PET in identifying hypoxic, radioresistant tumour regions [7]. These findings suggest that strategies which enhance the tumour radiosensitivity throughout the tumour volume, rather than focusing on sub-volumes, might improve outcomes for HPV − HNSCC.

FDG-PET imaging utilises [^18^F] fludeoxyglucose, a glucose analogue that accumulates in tumours due to overexpression of glucose transporters (GLUT1/2) [8, 9]. This increased demand for glucose facilitates glycolytic ATP production, even in the presence of oxygen; a metabolic adaptation commonly known as the Warburg effect, acknowledged as a hallmark of cancer [10–12]. Recent research has questioned whether metabolic vulnerabilities, such as dependency on monosaccharides, can be exploited to overcome treatment resistance. Screening of monosaccharides (e.g., galactose, fructose, fucose, glucose, and mannose) showed mannose in particular impairs tumour cell proliferation [13, 14]. Although internalised via the same transporters as glucose, mannose disrupts glycolysis, appearing to reduce lactate and pyruvate production [15]. Additionally, mannose enhanced tumour cell death when combined with chemotherapy drugs including cisplatin and doxorubicin [15, 16]. Mechanistically, the combination of mannose and cisplatin led to a reduction in anti-apoptotic proteins (Mcl, Bax, and Bcl-2), resulting in activation of the intrinsic apoptotic pathway through release of cytochrome-c [16, 17].

When internalised, mannose and glucose undergo independent metabolism, converging on the pyruvate intermediatory, fructose-6-phospate (Fru-6-P). Initially, glucose and mannose are converted by hexokinase enzymes to glucose-6-phosphate (Glu-6-P) and mannose-6-phosphate, respectively. Phosphoglucose isomerase then converts Glu-6-P to Fru-6-P, while phosphomannose isomerase (PMI) metabolises Man-6-P to Fru-6-P [18]. Studies suggest that lower PMI levels enhance mannose-driven metabolic reprogramming, potentially through the accumulation of toxic Man-6-P [15, 18, 19]. Interestingly, in murine embryonic development, PMI depletion proves lethal due to Man-6-P toxicity, exacerbated by mannose supplementation [19].

Intrinsically linked to therapeutic sensitivity is the DNA damage response (DDR), dependent on metabolic activity. Unrepaired DNA damage can lead to mutations and cell death, while functioning metabolic processes supply the energy required for DNA repair [20]. Key DDR signaling effectors, such as ATM and ATR kinases, regulate metabolism by modulating mTOR signaling and cellular energetics [21]. Additionally, DNA repair enzymes like PARP1 depend on NAD + metabolism, further emphasising the interplay between metabolic reprogramming, DNA repair and genomic stability [22]. Considering that the mechanism of action of cytotoxic chemotherapy agents such as cisplatin and radiotherapy converge on inducing DNA double strand break damage, we questioned if the metabolism altering properties of mannose could also be utilised to modulate the radiation response in HNSCC. This work aims to establish if disrupting mannose metabolism by supressing PMI function, beneficially accentuates the previously reported anti-tumour effects of mannose [23], highlighting PMI as a valid anti-tumour target.

## Materials and methods

### Cell culture and establishment of PMI KO cell lines

Human HPV- HNSCC cell lines (FaDu and CAL27) were obtained from ATCC (USA), and CAL33 cells from DSMZ (Germany). Cells were grown in Eagle’s minimum essential media (Gibco™) with 10% fetal bovine serum (Gibco™) at 37°C in a 5% CO₂ environment. Regular mycoplasma testing confirmed contamination-free cultures. Stable phosphomannose isomerase (PMI) knockout (KO)/ knockdown (KD) cell lines were created using PMI-targeted lentivirus (derived from the pLEentiCRISPRv2 plasmid, GeneScript) with CRISPR/Cas9 technology. The PMI guide RNA (gRNA) sequence used was GGCATACTGCTGCACCGCAC. Puromycin (A1113803, Gibco™) (1.25 µg/ml) selected successfully transduced cell colonies.

### Cell viability assays

Cells were treated with different mannose concentrations as per predefined schedules. After treatment, media was replaced with 10% Alamar Blue (Thermo Fisher) and incubated for 4 h. Fluorescence was measured using the FLUOstar Omega plate reader (excitation/emission: 544/590 nm), and viability calculated as per manufacturer guidelines.

### Growth curves

Cells were exposed to 20 mM mannose for 96 h. Every 24 h, cells were harvested, resuspended in medium, and counted. Live cells were confirmed using 0.4% trypan blue (Invitrogen, USA) and counted using the Countess II Cell Counter (Invitrogen).

### Clonogenic survival assays

HNSCC cells were seeded in triplicate in six-well plates and exposed to ionising radiation (IR). Normoxic assays included 0–8 Gy doses, while hypoxic assays (0.5% O₂) used 0–12 Gy, administered via an X-ray cabinet source (Faxitron CP-160). After 14 days, colonies were fixed and stained with 0.4% crystal violet (Sigma) in 70% methanol (Sigma) and then counted. Colonies with ≥ 50 cells were scored, and plating efficiency (PE) was calculated. Survival fraction (SF) was determined by dividing the PE of treated groups by control PE. Clonogenic survival data were fitted to the linear quadratic model: S(D) = exp(-αD-βD^2^), normalised to non-irradiated controls for each group.

### Protein extraction and Western blot analysis

Cells were washed with cold PBS and lysed in radioimmunoprecipitation assay (RIPA) buffer (Pierce) containing 1% protease and phosphatase inhibitors (Thermo Fisher). Protein concentrations were measured using the Bradford assay (Thermo Fisher). Hypoxic protein samples were collected within an InvivoO2 hypoxia workstation (Baker Ruskinn) after a minimum 4 h incubation. Proteins were separated via sodium dodecyl-polyacrylamide gel electrophoresis (Invitrogen) and transferred to nitrocellulose membranes (Amersham Protran). Membranes were blocked in 5% semi-skimmed milk (Milipore) for 1 h, then incubated overnight at 4°C with primary antibodies (1:1000 dilution): anti-Mpi (B-2) (sc-393484, Santa Cruz Biotechnology), anti-HIF-1α (EPR16897, ab179483, Abcam), and anti-alpha-tubulin (#2144, Cell Signaling). Anti-GAPDH (EPR16891, ab181602, 1:2500, Abcam) served as a loading control. HRP-conjugated secondary antibodies (1:5000, Thermo Fisher) and HRP-chromogen reagent (Thermo Fisher) enabled protein detection. Images were acquired using a gel-doc system (UVI Tech), then quantified with ImageJ for densitometry analysis.

### Reverse transcription quantitative PCR (RT-qPCR) analysis

Total RNA was extracted from isogenic FaDu and CAL33 cells using the Maxwell RSC simplyRNA (AS1390, Promega, USA) kit. RNA concentration and purity was assessed by Nanodrop spectrophotometry (Thermo Fisher Scientific), with 1 µg of total RNA used for cDNA synthesis. Reverse transcription was performed using the RevertAid First Strand cDNA Synthesis Kit (Thermo Fisher) in a thermocycler using the following conditions: 25 °C for 10 min, 42 °C for 60 min, and 85 °C for 5 min. The resultant cDNA was stored at − 20 °C until required.

Quantitative RT-PCR (qRT-PCR) was performed using a Roche LightCycler with SYBR Green I Mastermix (Roche, Switzerland). PCR cycle conditions were as follows: activation at 95 °C for 15 min, followed by 50 cycles of amplification (95 °C for 30 s, 58 °C for 10 s, 72 °C for 10 s). Fluorescence measurements were recorded at the end of each cycle, and primer specificity confirmed by the presence of a single melt curve peak. All values were normalised against the housekeeping gene RPL13A using the 2^−ΔCT^ method for relative quantification. All primers were obtained from Thermo Fisher using the following sequences:

RPL13A:

Forward:5’-TGGTCGTACGCTGTGAAGG-3’; Reverse: 5’-AGGAAAGCCAGGTACTTCAACTT-3’.

PDK1:

Forward:5’-AGTTCATGTCACGCTGGGTA-3’; Reverse: 5’-CAGCTTCAGGTCTCCTTGGA-3’.

CAIX:

Forward:5’-AGTTGCTGTCTCGCTTGGAA-3’; Reverse: 5’-TCGGAAGTTCAGCTGTAGCC-3’.

### 2’,7’-Dichlorodihydrofluorescein Diacetate (DCFH-DA) ROS assay

HNSCC cells were pre-treated with mannose for 24 h before radiation. Ten minutes post-radiation, cells were washed in phenol red-free media, incubated with DCFH-DA solution for 45 min at 37 °C in the dark, and fluorescence levels of ROS converted DCFDA measured (Ex/Em 485/535 nm). Relative ROS levels were normalised against the irradiated control. (DCFDA / H2DCFDA - Cellular ROS Assay Kit obtained from abcam, ab113851).

### 53BP1 Immunofluorescence

HNSCC cells treated with mannose for 24 h were irradiated (1 Gy), then fixed either 2 or 24 h later using 4% formalin (Sigma – 10 min), and permeabilised in 0.1% Triton-X 100 (Sigma – 10 min). After blocking with BSA-PBST, cells were stained with primary 53BP1 antibody (#4937, 1:1000, Cell Signaling) and Alexa Fluor 488 secondary antibody (ab150077, Abcam) at room temperature for 1 h. Slides were mounted with DAPI-containing medium (Fluoroshield, Abcam) and stored at 4 °C. Quantification of 53BP1 foci was performed using fluorescence microscopy (Olympus, CytoViva & Prior Scientific) on at least 50 cells per replicate.

### ATP assay

Cells were exposed to mannose for 24 h under normoxia (21% O₂), then transferred to a hypoxic chamber (0.5% O₂) for 4 h. After treatment, cells were counted, lysed with detergent under agitation (the ATP assay kit, ab83355, abcam), and incubated with ATP substrate solution. Fluorescence was measured (Ex/Em 535/587 nm) using a 96-well plate. ATP levels were normalised against untreated controls and quantified using a standard curve.

### Succinate assay

Mannose-treated cells were transferred to a hypoxic chamber (0.5% O₂) for 4 h, homogenised in succinate dehydrogenase buffer (SAB) (succinate assay kit, ab204718, abcam), and centrifuged (400 rcf, 10 min). Supernatants were filtered through a 10 kDa spin column (ab93349, Abcam) and diluted to a final volume of 120 µl in SAB. Samples (50 µl) were mixed with reaction buffer and incubated at 37 °C for 30 min in the dark. Absorbance at 450 nm was measured, and intracellular succinate levels were normalised against controls using a standard curve.

### Seahorse extracellular flux analysis

Mannose-treated HNSCC cells were analysed for bioenergetic function using a Seahorse Extracellular Flux Analyzer (Seahorse Bioscience). Cells (2 × 10^4^) were seeded in XF 96-well plates and incubated overnight. After washing, cells were maintained in XF assay medium with glucose, sodium pyruvate, and L-glutamine (Sigma). Baseline oxygen consumption rate (OCR) and extracellular acidification rate (ECAR) were recorded before mannose administration via automated injection. Changes in OCR and ECAR were monitored over 600 min, and phenograms were generated from readings at 300 min to compare bioenergetic profiles.

### In vitro metabolite extraction

Cells were treated with 20 mM mannose for 48 h +/- radiation (4 Gy), with radiation treatment 24 h post mannose addition. For stable isotope labelling, cells were cultured in glucose-free medium containing unlabelled glucose (5.5 mM), mannose (20 mM) or uniformly labelled ^13^C-glucose (^13^C_6_-Glc – 5.5 mM, Sigma) for 24 h to reach steady-state labelling. Five replicates were used per treatment condition. After treatment, media was removed, and cells were washed with ice-cold PBS before adding 1 ml of ice-cold metabolite extraction buffer (5:3:2 methanol: acetonitrile: ultra-pure water). Cells were scraped, transferred to cold 1.5 ml tubes, and incubated at 4 °C for 15 min at 800 rcf on a rocking platform. Samples were then centrifuged at 15,800 rcf for 15 min at 4 °C. The metabolite extract supernatant was collected, with both extracts and cell pellets stored at -80 °C. Extracts were dried under vacuum at 30 °C using an EppendorfTM Concentrator Plus and stored at -80 °C for further processing.

#### LC-MS data acquisition

Dried extracts were reconstituted in 110 µl mobile phase B and filtered through 0.2 μm PTFE filters at 10,000 rcf for 5 min. A 50 µl aliquot was transferred into deactivated autosampler vials for analysis. Metabolites (2.5 µL) were separated using a 15 min gradient from 70 to 100% mobile phase B (mobile phase A: 10 mM ammonium acetate in water, pH 9; mobile phase B: 10 mM ammonium acetate in 90% acetonitrile (v/v) in water, pH 9). A flow rate of 0.5 ml/min through an InfinityLab Poroshell 120 HILIC-Z column (2.1 × 100 mm, 2.7 μm) on an Agilent G7167B multisampler and G7120A pump was used. The gradient profile was: 0.0–11.5 min, 70% B; 11.5–12.5 min, 60% B; 12.5–13.5 min, 100% B; 13.5–15.0 min, and 100% B. The mass spectrometer (Agilent Dual ESI G6545B quadrupole time-of-flight (Q-ToF)) was operated in negative ion mode. Data acquisition and analysis were performed using Agilent MassHunter Qualitative Analysis and Profinder software. An internal library of compound standards was injected at regular intervals and used as references for sample data.

### Xenograft tumour models

Male SCID mice (CB17/lcr-Prkdcscid/lcrlcoCrlBltw, 5–6 weeks old) were obtained from Charles River, adhering to ethical standards of the QUB Animal Welfare and Ethical Review Body (AWERB), PREPARE guidelines and UK Home Office regulations. All experiments were performed in accordance with the approved protocol for experiment JC_2023_01 under Project Licence PPL2918. For tumour grafting, 2 × 10⁶ cells in Matrigel (Corning) were injected subcutaneously into the rear dorsum. Animals were monitored bi-weekly until tumour grafting occurred, with body weight recorded. Tumour volumes were calculated as length × width × height × 0.523 using calliper measurements. Upon reaching 80–120 mm³, mice were randomly assigned to treatment groups (minimum 8 per group). Mannose (200 µM) was administered daily for seven days by oral gavage (200 µl of 1 M mannose), and through drinking water supplemented with 0.5 M mannose. Tumour volumes were measured three times per week, with the experimental endpoint set at four times the initiation volume (maximum tumour burden = 480 mm³ ≡ 9.7 mm geometric mean diameter (GMD) – maximum permitted – 12 mm GMD), ensuring permitted limits were not exceeded.

### Tumoursphere assays

Cells were cultured in tumourspheres medium in ultra-low attachment 96-well plates (Corning). After 3 days, tumourspheres were treated with mannose and radiation (6–9 Gy) and returned to the incubator. Growth was measured three times per week, and media replenished using a 50% replacement method. Tumoursphere diameter was measured using the Cell3 Imager (SCREEN, Japan). Fluorescent imaging was performed on tumourspheres with a standardised diameter (600 μm), co-stained with a live-cell hypoxia marker (Sigma Aldrich) and Hoechst dye (Life Sciences) to visualise the nuclear compartment.

### Statistical analysis

Statistical analyses were performed in GraphPad Prism, with data expressed as mean ± SD (or S.E.M. for animal experiments). Clonogenic assays were analysed using two-way ANOVA with Tukey’s multiple comparisons test. All other experiments used one-way ANOVA with Tukey’s or Dunnett’s multiple comparisons tests. A p-value ≤ 0.05 was considered statistically significant.

## Results

### PMI depletion enhances the anti-tumour potential of mannose

**Fig. 1:**
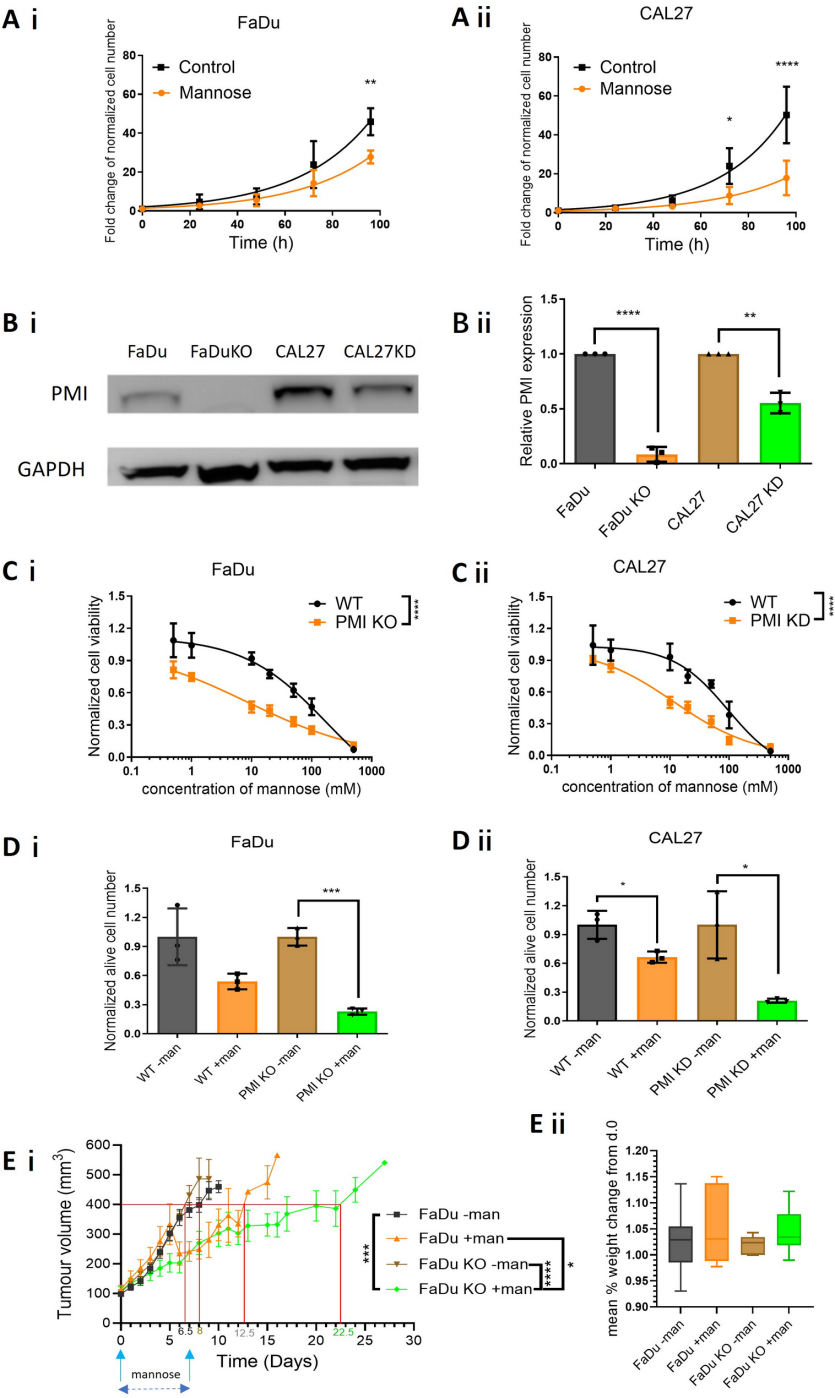
PMI depletion enhances the anti-proliferative effect of mannose. **A i & ii**) Growth curves of FaDu and CAL27 WT cells following exposure to mannose (20 mM). **B i & ii)** PMI protein expression in FaDu and CAL27 WT and KO/KD cells, quantification of relative PMI expression by densitometry analysis. **C i & ii)** Concentration dependent (0 mM – 500 mM) impact of mannose on cell viability in PMI WT and KO/KD FaDu and CAL27 cells, measured 48 h post-treatment using Alamar blue reagent. **D i & ii)** Trypan blue exclusion assay demonstrating increased sensitivity to mannose (20 mM) following PMI KO over 48 h. **E i)** Assessment of the direct anti-tumour impact of mannose in WT and KO FaDu xenograft tumours (*n* = 8 mice for each group). Animals bearing established tumours (100 mm^3^) were administered 200 µmol mannose (200 µl of 1 M mannose) via oral gavage, daily for seven days. Additionally, drinking water was supplemented with 10% mannose (0.5 M). Tumour volume was measured and recorded three times weekly, plotting mean tumour volume +/- SEM. **E ii)** Mean percentage weight change compared relative to experimental starting weight +/-SEM. Data presented (panels A-D) are mean ± SD of three independent biological replicates. *p ≤ 0.05 - Two-way ANOVA -Bonferroni’s multiple comparisons test and unpaired Student t-test

Mannose, a natural monosaccharide, is reported to inhibit tumour cell proliferation in several cancer models [14](Jin et al., 2023). Using three HPV- HNSCC models (FaDu, CAL27, CAL33), we observed significant (FaDu: *p* = 0.0028, CAL27: *p* < 0.0001) reductions in proliferation after prolonged mannose exposure (20 mM, 96 h). Mannose extended mean doubling times by 1 h and 3 h in FaDu and CAL27 cells respectively (Fig. 1A i & ii – CAL33 Fig. S1A). While basal phosphomannose isomerase (PMI) expression varied between models, it did not correlate with basal mannose sensitivity (IC_50_ range: 82–98 mM, Table 1). CRISPR/Cas9-mediated PMI knockout/down (KO/KD) lines revealed that PMI KO reduced protein expression by > 90% in FaDu (Fig. 1B i & ii) and CAL33 (Fig. S1B i & ii) cells, while CAL27 showed ~ 50% reduction, likely due to single-allele deletion (Fig. 1B i & ii). All PMI-suppressed cells displayed increased mannose sensitivity (Fig. 1C, Fig. S1C, Table 1). IC_50_ values in PMI KO FaDu and CAL33 cells were ~ 20-fold lower than wild-type (WT) lines (e.g., FaDu WT IC_50_: 176 mM; KO: 7.8 mM). CAL27 partial KD cells were 8-fold more sensitive, effects confirmed using trypan exclusion assays at 20 mM mannose (Fig. 1D, Fig. S1D).

**Table 1:** IC_50_ concentrations of mannose in WT and PMI knockout FaDu, CAL27 & CAL33 cells following a 48 h treatment period

Cell line	IC_50_ (mM)	95% CI (M)	*P*-value (vs. Parental)
FaDu	175.8	0-0.7	Reference
FaDu KO	8.7	0-0.03	< 0.0001
CAL27	95.85	0-0.21	Reference
CAL27 KD	11.82	0.002–0.021	< 0.0001
CAL33	100.3	0.013–0.18	Reference
CAL33 KO	5.194	0-0.014	< 0.0001

Subcutaneous FaDu WT and PMI KO tumours were grafted to assess systemic anti-tumour effects of oral mannose (Fig. 1E i). Without mannose, WT and PMI KO tumours grew at similar rates (endpoints: 6.5 vs. 8 days), indicating that PMI depletion alone does not affect tumour growth. Mannose treatment extended experimental endpoint in WT tumours by 4.5 days. Critically, PMI KO combined with mannose nearly doubled growth delay to 22.5 days (*p* < 0.0001), representing a 3.5-fold increase compared to untreated PMI KO tumours. Importantly, Neither PMI ablation nor mannose significantly affected mean body weight, suggesting good biocompatibility (Fig. 1E ii). These findings demonstrate that suppressing PMI activity significantly enhances the anti-tumour effect of mannose.

### Global metabolic suppression following mannose and PMI depletion

**Fig. 2:**
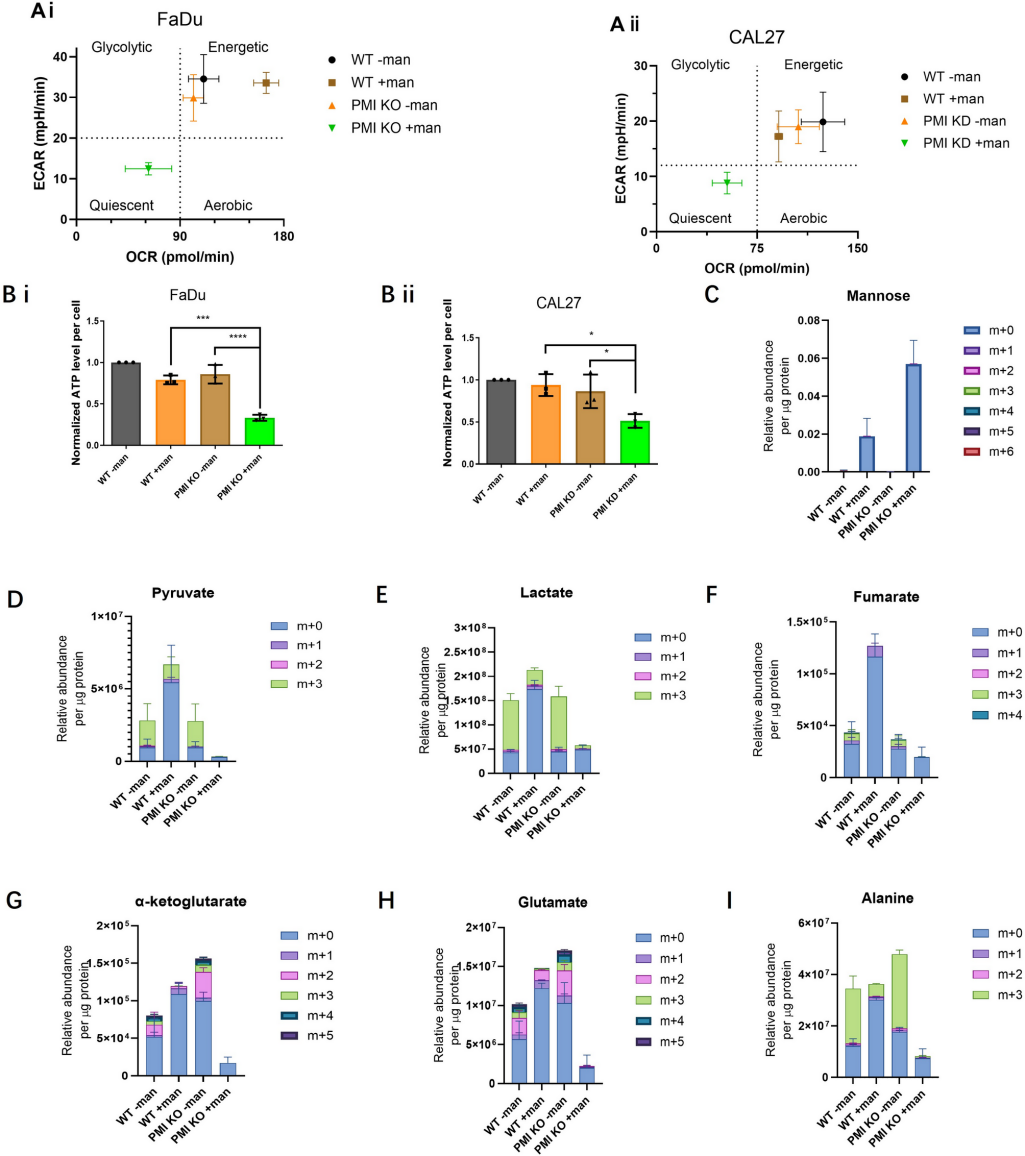
Metabolic shifts in glycolysis and oxidative metabolism induced by mannose following PMI depletion. Differential phenotypic profiles of WT and PMI KO/KD **A i)** FaDu and **A ii)** CAL27 cells 300 min post-mannose exposure assessing alterations to extracellular acidification (ECAR) and oxygen consumption (OCR) obtained through Seahorse analysis. LC-MS metabolite analysis using uniformly labelled ^13^C_6_-Glc in WT and PMI KO FaDu cells treated with or without mannose. **B)** Intracellular ATP levels per cell in WT and KO/KD models of **(i)** FaDu and **(ii)** CAL27 cells in the presence or absence of mannose treatment (24 h). **C-I)** Quantitative analysis of the relative abundance of key intracellular metabolites, including **C)** mannose, **D)** pyruvate, **E)** lactate, **F)** fumarate, **G)** a-ketoglutarate, **H)** glutamate and **I)** alanine (*n* = 4 or 5 per group). Data presented (panels A & B) are mean ± SD of three independent biological replicates. *p ≤ 0.05 - One-way ANOVA – Dunnett’s multiple comparisons test for **A)** and Tukey’s multiple comparisons test for **B)**

Using the Seahorse XF96 Analyser, we evaluated the impact of mannose and PMI depletion on metabolic function by measuring oxygen consumption rate (OCR) and extracellular acidification rate (ECAR), respectively reflecting cellular respiration and glycolysis. Neither PMI ablation nor mannose alone consistently altered OCR or ECAR in FaDu and CAL27 WT cells. However, the combination of PMI ablation and mannose significantly suppressed both OCR and ECAR, effectively collapsing energy production, inducing a quiescent like state (Fig. 2A i - FaDu OCR *p* < 0.0001, ECAR *p* < 0.0001; Fig. 2A ii CAL27 OCR *p* < 0.0001, ECAR *p =* 0.0006). This metabolic suppression corresponded to an 80% reduction in ATP levels in FaDu cells (*p* < 0.0001) and 50% in CAL27 (*p* = 0.0362), while neither mannose alone nor PMI KO/KD affected ATP levels (Fig. 2B i & ii).

To investigate mannose-driven changes in glucose metabolism, liquid chromatography - mass spectrometry (LC-MS) metabolomic analysis was performed on FaDu cells using uniformly labelled ^13^C_6_-Glucose (^13^C_6_-Glc). Cells were cultured in 5.5 mM ^13^C_6_-Glc for 24 h, allowing steady-state metabolite exchange before extracting and quantifying intracellular metabolites. Only mannose-treated cells exhibited detectable mannose, with PMI knockout (KO) FaDu cells accumulating nearly three times more mannose than WT cells (Fig. 2C), consistent with impaired mannose metabolism due to defective PMI activity. To assess the impact of mannose treatment and PMI ablation on glucose metabolism, glucose labelling of glycolytic intermediates was conducted. Similar to prior studies in osteosarcoma and pancreatic tumour models [13], mannose treatment in WT FaDu cells reduced glucose-derived pyruvate and lactate (m + 3 isotopologues) (Fig. 2D and E), while increasing overall metabolite abundance. This indicates mannose itself fuels glycolysis while blocking glucose-derived glycolytic flux. The absence of changes in extracellular acidification rate (ECAR) with mannose treatment (Fig. 2A) suggests that lactate generated from mannose-derived pyruvate is not exported. PMI KO alone had little impact on glucose-fuelled glycolysis, as shown by similar levels of m + 3 pyruvate and lactate (Fig. 2D and E). However, mannose treatment in PMI KO cells significantly reduced pyruvate and lactate abundance, independent of source, indicating severe glycolytic suppression, a result corroborating the extracellular flux data.

Besides producing lactate, pyruvate also feeds the TCA cycle via pyruvate dehydrogenase (PDH) and pyruvate carboxylase (PC) activity. We next examined the impact of treatment conditions on TCA cycle metabolite abundance, specifically fumarate and a-ketoglutarate (aKG). Both m + 2 and m + 3 isotopes of aKG and m + 3 fumarate were present in WT FaDu cells, suggesting PDH and PC activity. Mannose treatment blunted glucose-derived fumarate (m + 3) (Fig. 2F), but increased total fumarate abundance, suggesting mannose-derived pyruvate enters the TCA cycle. A similar trend was observed for α-ketoglutarate (aKG) (Fig. 2G), where mannose reduced m + 2 and m + 3 labelling but increased overall levels. This data, for the first time, shows that mannose treatment has metabolic impacts beyond the glycolytic cascade. PMI KO alone had minimal effects on fumarate but increased total aKG and m + 2 labelling, indicating distinct metabolic changes. Notably, these trends extended to glutamate (Fig. 2H), suggesting that PMI ablation affects pathways associated with glutamate metabolism. This includes glutamine-pyruvate transaminase (GPT), which catalyses aKG transamination with alanine to produce glutamate and pyruvate [24]. Alanine labelling (m + 3) from glucose remained unchanged (Fig. 2I), but overall abundance mirrored aKG and glutamate, pointing to alterations in transamination activity. However, most notably, across all examined metabolites, PMI KO cells treated with mannose exhibited profound reductions in abundance. Fumarate dropped by 50% (Fig. 2F) and aKG by 88% (Fig. 2G) compared to WT controls, with similar magnitude reductions in glutamate and alanine (Fig. 2H and I). Aspartate, a crucial part of the aspartate-malate shuttle, showed comparable declines (Fig. S2), suggesting impaired aspartate-glutamate conversion. These findings confirm that mannose treatment amplifies metabolic suppression across multiple pathways, particularly when PMI activity is impaired.

### Prolonged mannose metabolic pressure increases radiation sensitivity

**Fig. 3:**
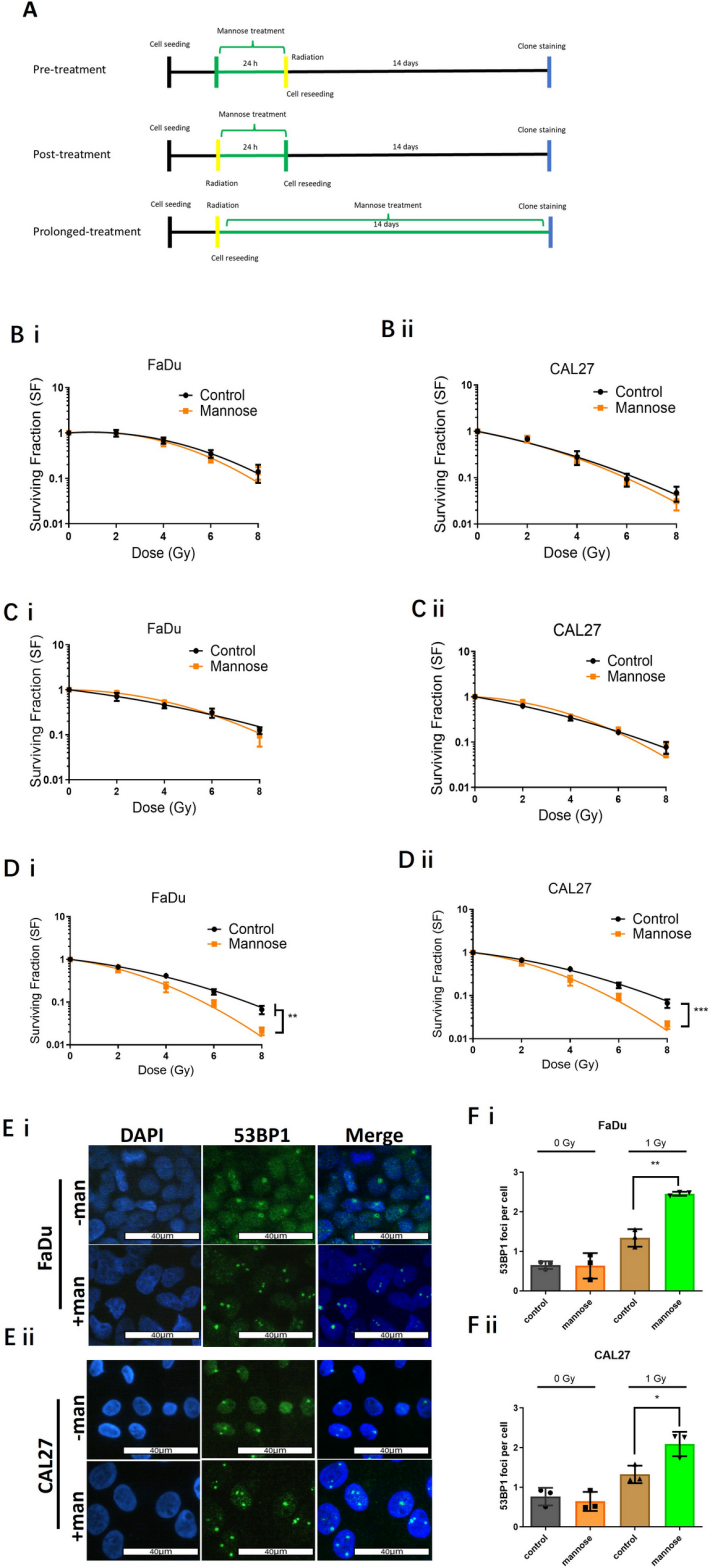
Radiation response of WT HNSCC tumour models to radiation. **A)** The effect of mannose treatment schedules on clonogenic survival assays. Treatment conditions include: pre-treatment - mannose (20 mM) 24 h prior to radiation; post-treatment - mannose (20 mM) 24 h after radiation treatment; or prolonged-treatment - mannose (20 mM) supplementation throughout the 14-day colony forming period. **B)** Pre-treatment clonogenic survival data for **(i)** FaDu and **(ii)** CAL27 WT cells +/- mannose. **C)** Post-treatment clonogenic survival data for **(i)** FaDu and **(ii)** CAL27 WT cells +/- mannose. **D)** Prolonged treatment (14 day) clonogenic survival data for **(i)** FaDu and **(ii)** CAL27 WT cells +/- mannose. **E)** Immunofluorescence images of 53BP1 foci, a surrogate indicator of DNA double strand break damage in WT **(i)** FaDu and **(ii)** CAL27 cells treated with mannose (20 mM) for 72 h prior to radiation (1 Gy), then fixed 24 h post-radiation allowing repair. **F)** Quantified residual DNA DSB damage 24 h post radiation (1 Gy) treatment in WT **(i)** FaDu and **(ii)** CAL27 cells. Foci were calculated by scoring a minimum of 50 cells per replicate, scored from three independent biological replicates (*n* = 3). Scale bars: 40 μm. Data presented (panels B-F) are mean ± SD of three independent biological replicates. *p* ≤ 0.05 - two-way ANOVA and unpaired Student t-test

To investigate whether mannose-mediated metabolic reprogramming enhances radiation sensitivity, colony-forming assays were performed under different mannose treatment schedules: 24 h pre- or post-radiation and prolonged exposure throughout the assay (~ 14 days - Fig. 3A). Interestingly, short-term mannose treatment (pre- or post-radiation) did not significantly alter radiation sensitivity (Fig. 3B and C). However, prolonged exposure significantly increased the sensitiser enhancement ratio (SER) in WT FaDu (SER: 1.28, *p =* 0.001) and CAL27 (SER: 1.24, *p =* 0.0003) cells (Fig. 3D). In CAL33 cells, short-term mannose exposure had no effect (Fig. S3A i & ii), and long-term exposure produced a minimal SER increase (1.1; Fig. S3A iii, Table 2), likely due to poor colony-forming capacity.

**Table 2:** Sensitiser enhancement ratio (SER) of WT FaDu and CAL27 cells following pre-treatment, post-treatment and prolonged-treatment to mannose (20 mM) compared relative to no mannose controls

SER	FaDu	CAL27
Pre-treatment	1.03	1.02
Post-treatment	0.89	0.87
Prolonged-treatment	1.28	1.24

Immunofluorescent 53BP1 staining showed that 2 h post-radiation, mannose treatment increased DSB damage across all cell models (Fig. S3B i-iii). Residual unrepaired DSBs 24 h post-radiation were significantly elevated: 82% (*p =* 0.001) in FaDu cells (Fig. 3E and F i), 58% (*p =* 0.02495) in CAL27 (Fig. 3E and F ii), and 105% (*p =* 0.026) in CAL33 (Fig. S3B iv). These results show that sustained mannose exposure enhances radiation sensitivity by inducing metabolic reprogramming, increasing DNA damage, and impairing repair processes.

### PMI depletion promotes mannose mediated radiosensitisation

**Fig. 4:**
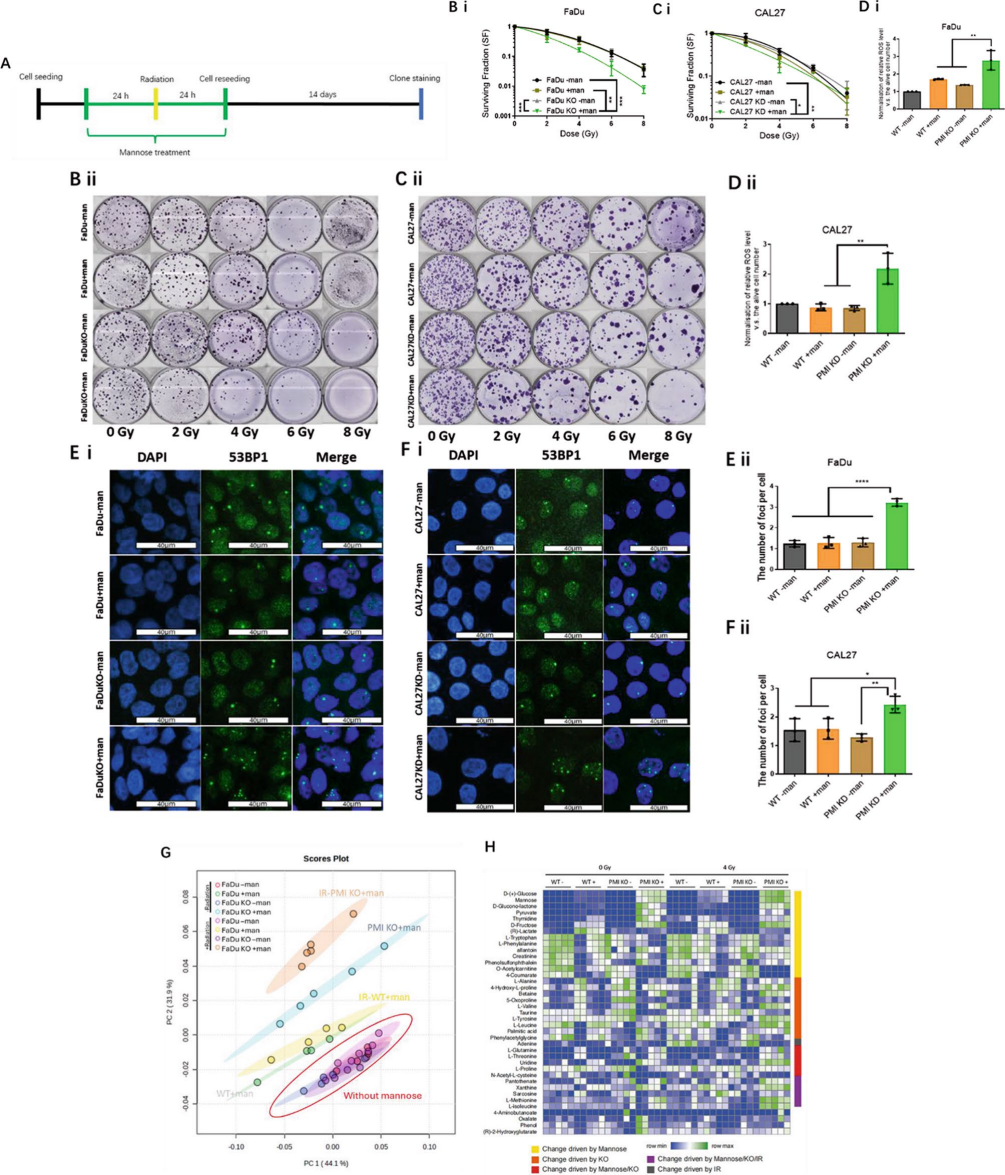
Radiosensitization in PMI ablated HNSCC models driven by combined PMI ablation and mannose mediated metabolic alterations. **A)** Schematic mannose treatment schedule of colony forming assay. Clonogenic assays in WT and PMI KO/KD **B i & ii)** FaDu and **C i & ii)** CAL27 cells, +/- mannose (20 mM) pre-treatment (48 h), followed by radiation (2–8 Gy). D) Relative ROS levels detected using 2’,7’-dichlorodihydrofluorescein diacetate (DCFH-DA) in WT and PMI KO/KD (i) FaDu and (ii) CAL27 cells +/- mannose (20 mM) pre-treatment (24 h) prior to radiation (4 Gy). ROS levels were measured 10 min post-radiation treatment. **E)** 53BP1 immunofluorescence in WT and PMI KO **(i)** FaDu cells with **(ii)** quantified differences calculated by scoring a minimum of 50 cells per independent replicate. Scale bars: 40 μm. **F)** 53BP1 immunofluorescence in WT and PMI KD **(i)** CAL27 cells with **(ii)** quantified differences calculated by scoring a minimum of 50 cells per independent replicate. Unlabelled LC-MS analysis of the metabolic profiles of WT and PMI KO FaDu cells treated with mannose, +/- radiation (4 Gy). Scale bars: 40 μm. **G)** Principal Component Analysis (PCA) depicting distinctive clustering of the metabolome in mannose exposed cells. **H)** A heatmap generated from thirty-nine dominant metabolites for each treatment group (*n* = 5 for each group). Data presented (panels B-F) are mean ± SD of three independent biological replicates. *p ≤ 0.05 - One-way ANOVA - Tukey’s multiple comparisons test **B-D)** and two-way ANOVA - Tukey’s multiple comparisons test **A)**

Colony-forming assays were performed in PMI-depleted models with mannose exposure limited to 48 h (24 h pre- and 24 h post-radiation, Fig. 4A). PMI loss without mannose had no significant effect on radiation sensitivity in FaDu or CAL27 cells (Fig. 4B & C). However, PMI KO/KD combined with short-term mannose exposure significantly increased radiation sensitivity, yielding mean SERs of 1.51 (*p =* 0.0003) and 1.35 (*p =* 0.003) in FaDu (Fig. 4B i & ii) and CAL27 (Fig. 4C i & ii), respectively. Additionally, key radiobiological data are included in Table 3, detailing the dose enhancement factor (DEF) at 2 Gy, selected due to alignment with a typical clinical dose per fraction, along with the α, β and α/β ratio, parameters extrapolated from the radiation survival curves.

**Table 3:** Radiation dose enhancement factor (DEF) @2 Gy from pre + post mannose treated FaDu and CAL27 cells under normoxia (Fig. 4A). The α/β ratio represents the dose where the contribution to cell death is equal between both linear and quadratic components extrapolated from the linear quadratic $\:SF={e}^{-(\alpha\:D+{\beta\:D}^{2})}$ fit

	FaDu	FaDu +mannose	FaDu KO	FaDu KO +mannose	CAL27	CAL27 +mannose	CAL27 KD	CAL27 KD +mannose
DEF @ 2GY	Reference	1.10	1.00	1.73	Reference	1.18	1.14	1.54
α	0.112	0.149	0.122	0.323	0.142	0.111	0.118	0.303
β	0.037	0.031	0.035	0.034	0.053	0.045	0.032	0.016
α/β	3.03	4.80	3.48	9.5	2.68	2.46	3.68	18.93

As reactive oxygen species (ROS) underpin radiation induced indirect effects, and mannose alters mitochondrial function (a major ROS source), ROS levels were assessed following 4 Gy irradiation. In the absence of IR, no significant increase in ROS was observed, however, we did note a small increase in ROS following mannose treatment, an effect most pronounced following PMI KO/KD (Fig. S4), likely triggered by mitochondrial stress following M-6-P accumulation and the subsequent suppression of glycolysis. However, PMI KO/KD plus mannose increased ROS by 2.5-fold (*p* < 0.0002) in FaDu (Fig. 4D i) and 2-fold (*p* = 0.002) in CAL27 (Fig. 4D ii) compared to controls. Using the same short-term mannose treatment (24 h pre- and 24 h post-radiation), 53BP1 foci repair post-IR were quantified in PMI-depleted cells. Similar to WT cells, unresolved 53BP1 foci (24 h post-IR) were elevated in mannose-treated PMI KO/KD cells to a greater extent than in wild-type cells, resulting in 2.5-fold (*p* < 0.0001) more unresolved DNA DSBs in FaDu (Fig. 4Ei & ii), with slightly smaller increases (*p* = 0.032) in CAL27 (Fig. 4Fi & ii). These data suggest that mannose-mediated radiosensitisation is partly due to impaired DNA damage repair, further exacerbated by PMI suppression.

To investigate if PMI ablation and mannose coupled with radiation further impacts metabolic responses, we quantified unlabelled intracellular metabolite abundance in FaDu cells (robust PMI KO) again using LC-MS. Principal component analysis (PCA) was used to assess global metabolic alterations. Without mannose, all treatment groups (WT, PMI KO, and +/- radiation) clustered together, indicating minimal metabolic alterations from PMI ablation (supporting data in Fig. 2), or radiation alone (Fig. 4G). Consistent with earlier data, mannose caused a significant metabolic shift, exacerbated by PMI KO, indicated by distinctive PCA clustering (Fig. 4G: WT + mannose = green; WT + mannose + IR = yellow; PMI KO + mannose = Blue; PMI KO + mannose + IR = Orange). IR (4 Gy) had minimal effects in WT cells treated with mannose (green vs. yellow), while combined with mannose and PMI KO (orange) resulted in further significant metabolic stress.

To identify if specific metabolic pathways underpinned PCA clustering, we analysed individual metabolite abundance via heatmaps, focusing particularly on PMI KO + mannose + IR responses. Trends from t-tests between treatment groups (e.g., mannose vs. control) were used to cluster metabolites (Fig. 4H). Mannose had a clear effect on a subset of metabolites (yellow cluster), as does PMI KO (orange cluster), with little overlap between groups. The combination of mannose and PMI KO often enhances the metabolic impact of mannose (red cluster), reinforcing earlier observations. A few metabolites were specifically altered by the combination of all three conditions, classed as “driven by mannose/KO/IR” (purple cluster) including L-isoleucine, methionine and betaine (Fig. S5), likely representing driver metabolites differentiating between PCA clustering of mannose/KO vs. mannose/KO/IR. Taken together, both PCA and heatmap clustering demonstrate that radiation further exacerbates the metabolic stress caused by combined mannose/PMI KO.

### Mannose mediated modulation of the response to hypoxia

**Fig. 5:**
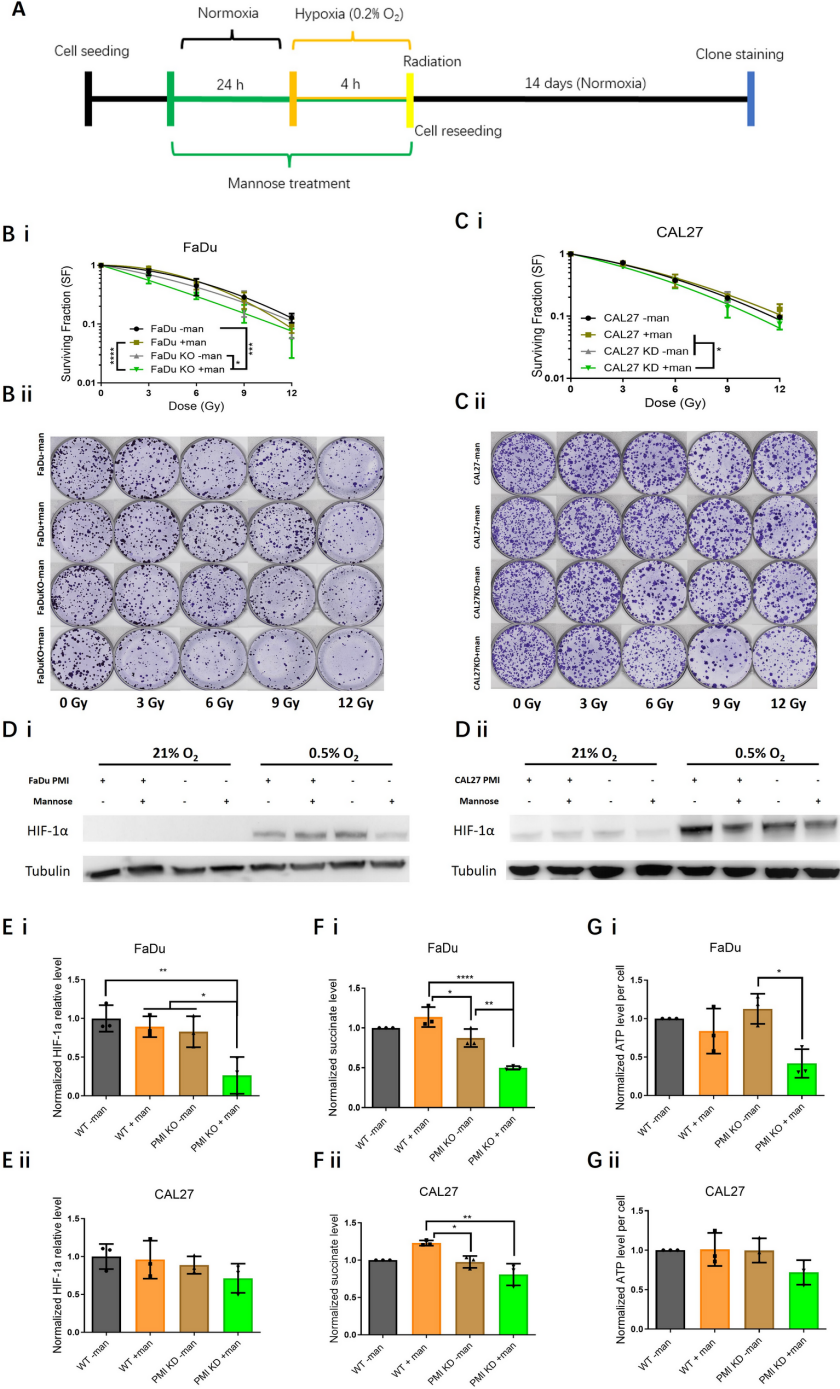
Overcoming hypoxia-Induced radioresistance through HIF-1α suppression by mannose and PMI Knockout. **A)** Schematic mannose treatment schedule of colony forming assay. Colony forming assays in WT and PMI KO/KD **B i & ii)** FaDu, and **C i & ii)** CAL27 cells +/- mannose (20 mM) for 24 h prior to radiation treatment (RT). Four hours before RT, cells were transferred to an InvivO_2_ hypoxic workstation set to 0.5% O_2_. **D)** Western blot and **E)** semi-quantitative analysis of relative HIF-1α levels in WT and PMI KO/KD cells. **D i & E i)** FaDu and **D ii & E ii)** CAL27 cells +/- mannose for 24 h, with a 4 h hypoxia incubation prior to sample collection. **F)** Relative levels of succinate in WT and PMI KO/KD **i)** FaDu and **ii)** CAL27 cells pretreated +/- mannose (20 mM) for 24 h, with a 4 h hypoxia incubation prior to sample collection. **G)** Relative ATP levels of WT and PMI KO/KD **i)** FaDu and **ii)** CAL27 cells pretreated +/- mannose (20 mM) for 24 h, with a 4 h hypoxia incubation prior to sample collection. Data presented (panels **B**,** C**,** E-G**) are mean ± SD of three independent biological replicates. *p ≤ 0.05 -One-way ANOVA - Tukey’s multiple comparisons test **E-G)** and two-way ANOVA - Tukey’s multiple comparisons test **B&C)**

Tumour hypoxia contributes to radiation resistance in HNSCC. Experiments were conducted under hypoxic conditions (< 0.5% O_2_) to assess the effects of PMI depletion and mannose on radiation sensitivity. Hypoxia caused significant radioresistance, with an oxygen enhancement ratio (OER) of approximately 2 in both FaDu (Fig. S6A) and CAL27 cells (Fig. S6B; Table S1). Mannose alone did not affect radiation sensitivity, but mannose supplementation 24 h prior to radiation treatment sensitised (*p* < 0.0001) hypoxic FaDu PMI KO cells (SER 1.39 – Fig. 5B) and CAL27 PMI KD cells (SER 1.14 – Fig. 5C - *p* = 0.04748) compared to WT cells supplemented with mannose, effects further enhanced at higher radiation doses.

We next investigated whether mannose altered HIF-1α levels, a key regulator of the hypoxic response. Basal HIF-1α levels were low or undetectable under normoxic conditions. Short-term hypoxia increased HIF-1α expression, with mannose treatment reducing HIF-1α by ~ 75% in both PMI KO FaDu cells (Fig. 5D and E i – *p* = 0.0062) and CAL33 KO cells (Fig. S6C – *p* = 0.0202), and to a lesser extent in CAL27 cells (Fig. 5D and E ii), relative to WT controls. To validate the downstream effects of mannose-induced HIF-1α suppression in PMI KO cells, qRT-PCR analysis was performed to assess alterations in HIF-1 target gene expression, specifically probing PDK-1 and CAIX (Fig. S6D&E). Results revealed a significant suppression of PDK-1 mRNA in FaDu KO cells, equating to a 85% suppression in PDK-1 mRNA compared to PMI KO cells without mannose (Fig. S6Di). Similarly, in CAL33 cells, a 68% reduction in PDK-1 expression was observed (Fig. S6Dii). For CAIX expression, the results were less conclusive. While CAIX levels were lower in PMI-KO mannose-treated CAL33 cells (Fig. S6Eii), no clear trend was observed in FaDu cells (Fig. S6Ei), irrespective of mannose treatment or PMI status. These findings indicate that while PDK1 expression is significantly affected by PMI depletion and mannose treatment leading to HIF-1α depletion, variances observed in CAIX may be more cell dependent.

Tumour cells rely on glycolysis under hypoxic stress to generate pyruvate, fuelling the TCA cycle to sustain intracellular ATP. TCA cycle intermediaries such as succinate, serve as metabolic indicators of mitochondrial activity under hypoxic stress. In mannose-treated PMI KO/KD cells, the observed decrease in succinate levels reflects TCA cycle suppression, consistent with impaired mitochondrial respiration and reduced OCR (Fig. 2A). Earlier we demonstrated that mannose reduces oxidative glycolysis (Fig. 2), here demonstrating similar biology under hypoxia, assessed by directly measuring succinate and ATP levels in response to mannose treatment. While succinate levels remained unchanged in WT cells, PMI KO/KD combined with mannose treatment led to a significant reduction in succinate levels by 50% in FaDu cells (WT + mannose: *p* < 0.0001; PMI KO: *p* = 0.0029) and 35% in CAL27 cells (*p* = 0.0013) (Fig. 5D i & Fig. 5F ii). This metabolic shift coincided with a marked reduction in ATP production, with mannose treated PMI KO/KD cells exhibiting a 59% decrease (*p* = 0.0102) in ATP levels under hypoxia (0.5% O_2_) in FaDu cells, and a 30% decrease in CAL27 cells (Fig. 5G i & ii). These results support a model in which mannose disrupts mitochondrial respiration, potentially increasing intracellular oxygen availability, thereby counteracting hypoxia-induced radioresistance.

### Mannose mediated 3D-tumoursphere radiosensitisation by supressing hypoxia

**Fig. 6:**
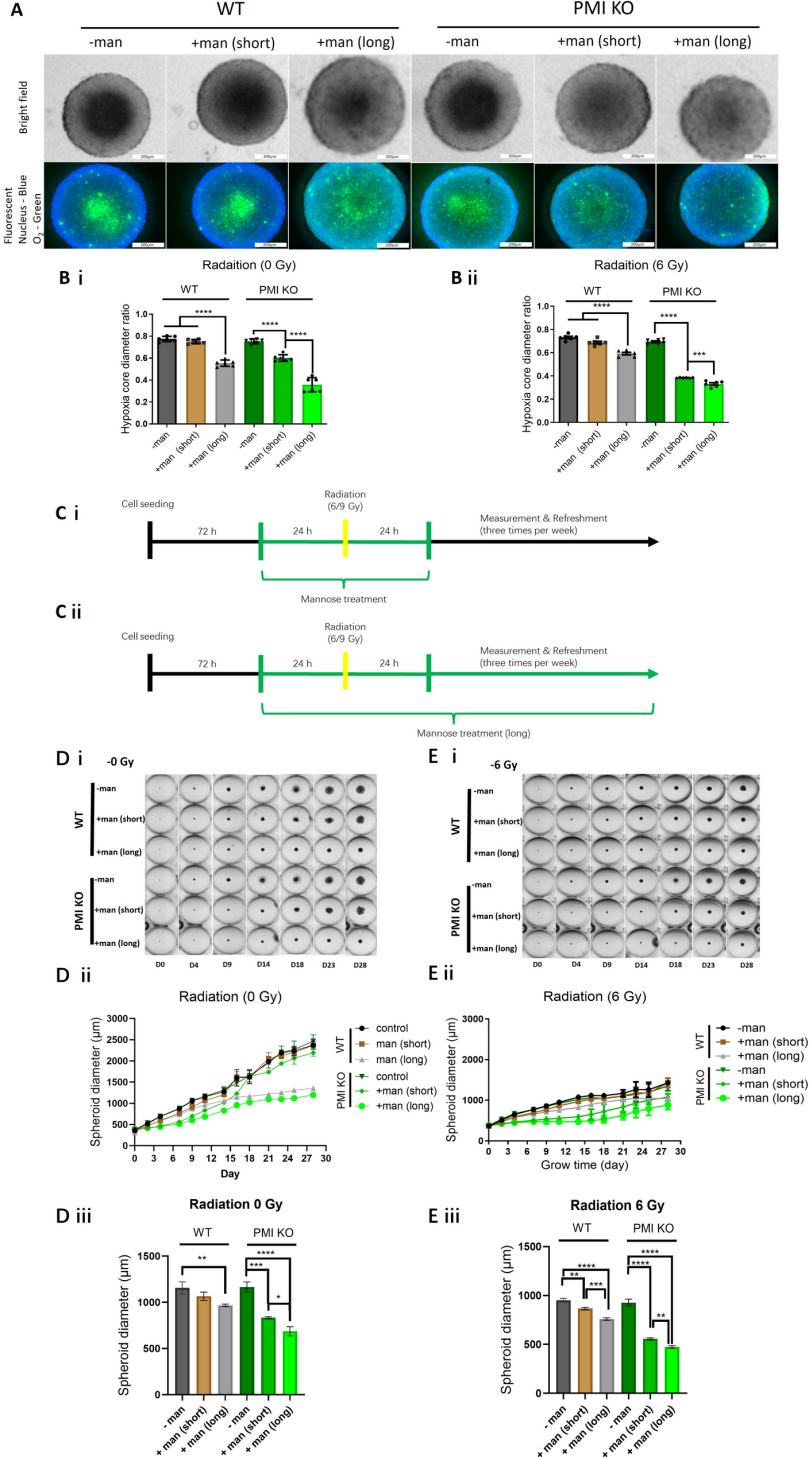
Mannose sensitises PMI KO HNSCC tumourspheres to radiation. **A)** Confocal fluorescence (Image-iT™ Green Hypoxia Dye and Hoechst staining) and brightfield imaging of size matched (~ 600 μm) FaDu (WT/KO) tumourspheres pre-treated with mannose (20 mM). **B)** Diameter ratio of the necrotic/hypoxic core in **(i)** unirradiated or **(ii)** 6 Gy irradiated FaDu (WT/KO) tumourspheres treated +/- mannose (20 mM for 48 h for short-term exposure and sustained exposure to mannose for long-term treatment). **C i & ii)** Schematic diagram illustrating treatment scheduling for both WT and PMI KO FaDu tumoursphere models. **D i)** Representative images of unirradiated WT and PMI KO FaDu tumourspheres exposed to short-term (48 h) and long-term (sustained) mannose treatment. **D ii)** Dynamic growth data of unirradiated WT and PMI KO FaDu tumoursphere models. **D iii)** Differential tumoursphere diameter on Day 11 for WT and PMI KO tumourspheres treated with mannose for short-term (48 h) or long-term (sustained). **E i)** Representative images of irradiated (6 Gy) WT and PMI KO FaDu tumourspheres with short-term (48 h) and long-term (sustained) mannose treatment. **E ii)** Dynamic growth data of unirradiated WT and PMI KO FaDu tumoursphere models. **E iii)** Differential tumoursphere diameter on Day 11 for irradiated WT and PMI KO tumourspheres treated with mannose for short-term (48 h) or long-term (sustained). Data are presented as mean ± SD of three independent biological replicates. *p ≤ 0.05 (One-way ANOVA- Tukey’s multiple comparisons test (B, D &E)

Tumourspheres mimic solid tumours with natural nutrient and oxygen gradients. Given that mannose promotes hypoxic radiosensitisation in 2D clonogenic assays, we tested whether mannose combined with PMI depletion could alter tumoursphere oxygenation and radiation sensitivity. Image-iT™ Green Hypoxia Dye, counterstained with Hoechst, was used to assess the impact of mannose on oxygen gradients within FaDu tumourspheres. Images were acquired based on matching tumoursphere diameter (~ 600 μm), rather than time post treatment, mitigating against size-limited diffusion (Fig. 6A). Image-iT™ Green Hypoxia Dye is an organic iridium complex possessing fluorescence properties quenched by oxygen. Hypoxic staining was prominent in both WT and PMI KO FaDu tumourspheres, with no change after short-term mannose exposure in wild-type models. The relative ratio of green Image-iT fluorescence compared against Hoechst fluorescence remained unchanged across WT FaDu tumourspheres irrespective of mannose treatment. However, in PMI KO FaDu tumourspheres, short-term mannose treatment resulted in an approximate 50% reduction in the fluorescence ratio, while sustained mannose led to a > 90% reduction (Fig. S7A). Additionally, long-term mannose exposure, even in WT FaDu tumourspheres, reduced fluorescence intensity indicating an increase in tumoursphere oxygenation, particularly within the tumoursphere core. In PMI-depleted tumourspheres, fluorescence patterns were similar to wild-type, with prolonged mannose exposure largely eliminating low-oxygen staining. Quantifying the hypoxic core ratio (defined as the optically dense/dark core - Fig. 6A&B) revealed that prolonged mannose exposure significantly (*p* < 0.0001) reduced the hypoxic core by 22% in WT FaDu tumourspheres. In PMI KO tumourspheres, short-term mannose reduced the hypoxic ratio by 15%, increasing to 40% with prolonged exposure (Fig. 6B i). Overall, the magnitude of effect was similar following radiation (6 Gy – Fig. 6B ii), except that the hypoxic core following short-term mannose treatment was further significantly reduced, decreasing by 25% over equivalent tumourspheres without radiation.

We next assessed the effects of mannose and PMI depletion on tumoursphere growth over 30 days. Two exposure regimes were tested: short-term (24 h pre- and post-radiation − 48 h total) and long-term (mannose in media for 30 days, +/- radiation – Fig. 6C). Growth rates were compared between unirradiated controls (Fig. 6D) and tumourspheres treated with radiation (6 Gy - Fig. 6E Gy - Fig. S7C). FaDu tumourspheres followed Gompertzian growth kinetics, with significant (*p* < 0.001) growth inhibition in both unirradiated PMI WT and KO tumourspheres exposed to long-term mannose treatment, resulting in ~ 40% retardation of tumoursphere growth over 30 day (Fig. 6D ii). Conversely, short-term mannose had no impact on growth rates of unirradiated PMI WT tumoursphere growth, only significantly (*p =* 0.0001) reducing tumoursphere growth by 30% in the PMI-depleted background (Fig. 6D ii & iii). For radiosensitisation, the growth kinetics of mannose-treated tumourspheres were compared to PMI controls, normalising against radiation effects. In PMI functional tumourspheres, mannose (short or long term) showed similar effects to unirradiated controls. However, PMI-deleted tumourspheres without mannose were 1.64 (*p* = 0.0009) and 1.85 (*p* = 0.0003) larger than mannose-treated counterparts, an effect further enhanced compared to irradiated wild-type PMI tumourspheres (Fig. 6E ii & iii). Increasing radiation dose produced a similar magnitude of radiosensitisation, with maximal effects in PMI-depleted tumourspheres exposed to mannose (Fig. S7C i-iii). The direct impact of radiation on tumoursphere growth was also evident through the inverse correlation between radiation dose and tumoursphere diameter, observed in both WT and PMI KO groups by Day 11 (Fig. S7B). Finally, viability assays confirmed that radiation plus mannose did not simply kill PMI-deleted cells at the start of the assay, but instead impaired growth or induced quiescence (Fig. S7 D). These results show that mannose, particularly in PMI-depleted tumourspheres, significantly suppresses hypoxia and growth, enhancing radiation sensitivity.

## Discussion

This study investigates the metabolic effects of mannose, a sugar molecule that differs from glucose by the position of a single hydroxyl group. While subtle in its structural difference, the metabolic impact of mannose and glucose are not the same. Mannose has been shown to significantly alter biological processes, especially in the context of cancer metabolism [14, 25, 26]. Landmark work by Gonzalez et al. (2018) associated mannose supplementation with reduced tumour growth and increased sensitivity to cisplatin, sparking interest in the therapeutic potential of mannose [15]. The primary intracellular fate of mannose is linked to the expression of two key enzymes namely phosphomannomutase (PMM2) and phosphomannose isomerase (PMI), fulfilling respective roles in protein glycosylation or glycolytic catabolism [27, 28]. Recent studies report mannose mediated protection of the intestinal barrier lessening the effects of ulcerative colitis, to immune-metabolic reprogramming limiting the infective potential of common viruses, effects linked to the glycosylation role of mannose, with little direct impact on proliferation [29, 30]. Our data indicate a differential response, more likely common to tumour models. Mannose, even with functioning PMI dampens tumour cell proliferation, proving directly cytotoxic at mid-to-high mM concentrations (Fig. 1A and C). This effect was previously accredited to the accumulation of Man-6-P, impeding the enzymatic function of phosphoglucose isomerase (PGI), thereby blocking conversation of Glu-6-P to Fru-6-P, impairing glucose metabolism [30, 31]. Additionally, loss-of-function of PMI results in a futile cycle of mannose phosphorylation and dephosphorylation of Man-6-P, an effect known as the “honeybee effect,” responsible for bee lethality [19, 32]. Either way, sustained supraphysiologic levels of mannose following PMI depletion, markedly increased tumour cell sensitivity to mannose, by at least an order of magnitude in cells with ablated PMI (Fig. 1C and D). From a therapeutic perspective, irrespective of underpinning mechanism, increased sensitivity following partial knockdown of PMI highlights the potential of PMI as a therapeutic target in cancer. To illustrate this, the growth rate of FaDu isogenic (+/- PMI) xenograft tumours were halved by simply increasing mannose blood serum levels, without causing systemic toxicity (Fig. 1E).

To date, pre-clinical studies demonstrating anti-tumour effects mediated by mannose depend upon sustained metabolic pressure achieved though continual mannose supplementation [15, 33]. In the current study, short-term (i.e. within hours) global bio-energetic studies were conducted using the Seahorse Analyser. In this context, tumour associated oxidative glycolysis dominated all experimental groups, except for isogenic PMI compromised cells (Fig. 2A), underpinning our earlier observations of increased sensitivity to mannose following PMI deletion. Using ^13^C_6_-Glc, we probed deeper into the metabolic alterations following PMI ablation and mannose. LC-MS data confirmed that PMI KO resulted in mannose accumulation, with knock-on effects resulting in the loss of pyruvate, lactate, fumarate and subsequent downstream TCA cycle metabolites. These data indicate that PMI loss triggers an almost immediate metabolic stress following exposure to mannose that potently supresses tumour dependent glycolysis (Fig. 2D-G). Beyond glycolysis, loss of a-ketoglutarate, a key substrate for glutamate production via the activity of aspartate aminotransferase (AAT), drives knock-on implications in the translocation of electrons across the inner mitochondrial membrane, dampening electron transfer and ATP generation through oxidative phosphorylation, triggering global metabolic stress [34–36].

Evidence that mannose increased tumour cell sensitivity to cisplatin, a DNA damaging agent which forms covalent adducts with purine nucleobases, led us to ask if similar effects could be observed by combining mannose with radiation [15]. Interestingly, the duration of mannose exposure played a key role in influencing radiation sensitivity. Scheduling experiments using wild-type HNSCC indicate that short-term metabolic stress triggered by mannose prior-to or post-radiotherapy had no overall influence on radiation sensitivity, fitting with the conversion of Man-6-P to Fru-6-P, fuelling glycolysis and TCA activity in tandem with glucose. However, only in the context of sustained mannose exposure, likely resulting from the eventual accumulation of Man-6-P and the associated inhibition of phosphoglucose isomerase, does mannose significantly enhance radiation sensitivity (Fig. 3B-D) [19]. As DNA double strand breaks constitute the primary mechanism for radiation induced cell death, we probed the ability of mannose to influence both DSB yields and DNA damage repair, with significant increases in DNA DSB yields observed in all three cell lines tested, effects sustained for at least 24 h post radiation treatment (Fig. S3B & C and Fig. 3E&F).

Switching to a PMI deficient setting, short-term mannose exposure (24 h pre-RT and 24 h post-RT), previously failing to modulate radiation response, increased radiation sensitivity by up to 50%, effects coupled with elevated intracellular ROS and increased residual DNA damage (Fig. 4D-F). Importantly, these findings are corroborated by a previous study that reported mannose radiosensitisation under normoxia, effects most pronounced in esophageal squamous cell tumour models exhibiting low endogenous PMI expression [37]. Mechanistically, reports linked this response to impaired PMM2 glycosylation following prolonged mannose exposure, promoting proteasomal degradation of mRNAs linked to DNA damage repair, resulting in radiosensitisation [38]. Herein we demonstrate a glycosylation independent, complementary mechanism by which mannose radiosensitises HPV- HNSCC tumour models, attributed to impaired glycolytic flux.

Given that PMI ablation plus mannose efficiently sensitises to radiation, we questioned if this combination resulted in metabolic alterations beyond glycolytic suppression. Surprisingly, radiation alone had minimal direct impact, with no observable difference in the metabolome of FaDu cells (+/-PMI), a result in stark contrast following mannose treatment. While this points to the majority of the effect being driven by mannose, there remained distinctive separation and clustering of the metabolites following mannose and radiation treatment in the PMI depleted setting (Fig. 4G and H), indicating underpinning biology directly linked to increased radiation sensitivity (Fig. S5 A-F). Combination treatment supported the accumulation of the branched-chain amino acid L-isoleucine (Fig. S5 A), representing a potential salvage strategy as an alternative energy substrate, helping sustain DNA repair under conditions of glycolytic energy deprivation [39]. Interestingly, our findings also suggest that impaired mannose metabolism combined with radiation amplifies the inhibitory effects of radiation on one-carbon metabolism [40, 41]. This is evidenced by a significant accumulation of l-methionine and betaine (Fig. S5 B&C), key substrates of the methionine cycle, a pathway synonymous with high proliferative capacity in cancer [42]. Relating specifically to elevated nucleotide turnover, inhibited phosphoglucose isomerase (PGI) mediated by Man-6-P is reported to promote the pentose-pathway shunt through Glu-6-P accumulation [31, 43]. With an excess of 6-P-gluconolactone, eventually metabolised to ribose-5-P and phosphoribosyl diphosphate (PRPP), pre-cursors of *de novo* nucleotide synthesis, it is perhaps not surprising to find that mannose/radiation promotes pyrimidine synthesis (Fig. S5 D&E), evidenced by elevated thymidine and uridine [44–46].

The principal of “metabolic radiosensitisation” exploits drug induced inhibition of oxidative phosphorylation, supressing OCR and helping to counteract the effects of tumour hypoxia [47, 48]. Drugs specific to electron transport chain mitochondrial membrane complexes such as metformin and atovaquone, are reported to act as effective radiation sensitisers [49–51]. Given that mannose and PMI ablation contributed to OCR collapse, coupled with glutamate triggered inhibition of the malate–aspartate shuttle and oxidative phosphorylation, we queried if mannose/PMI ablation could help counteract hypoxia mediated radioresistance. Importantly, mannose supplementation significantly radiosensitised PMI KO/KD cells, with the magnitude of effect increasing with radiation dose (Fig. 5B and C). These effects were accompanied by reduced hypoxic HIF-1α expression (Fig. 5D and E), resulting in significant suppression of PDK-1, a key HIF-1 target gene (Fig. S6D&E), pointing to metabolic alterations that may overcome hypoxia driven resistance. Interestingly, this effect appeared to be driven by increased intracellular oxygen availability, resulting in HIF-1α destabilisation. Secondary to this response, reports have suggested that the loss of succinate, an outcome of supressed TCA cycle activity, promotes prolyl hydroxylases activity causing HIF-1α degradation [52]. While succinate levels were supressed in our studies (Fig. 5F), we hypothesise that the dominant effect is likely driven by decreased in oxygen consumption. This mechanism is further supported by the 3D tumoursphere models, where we observed increased oxygenation due to the loss of green fluorescence using the hypoxia responsive Image-iT™ probe, an effect further enhanced with prolonged mannose exposure, leading to supressed tumoursphere growth when combined with radiation (Fig. 6 and Fig S7).

## Conclusions

**Fig. 7:**
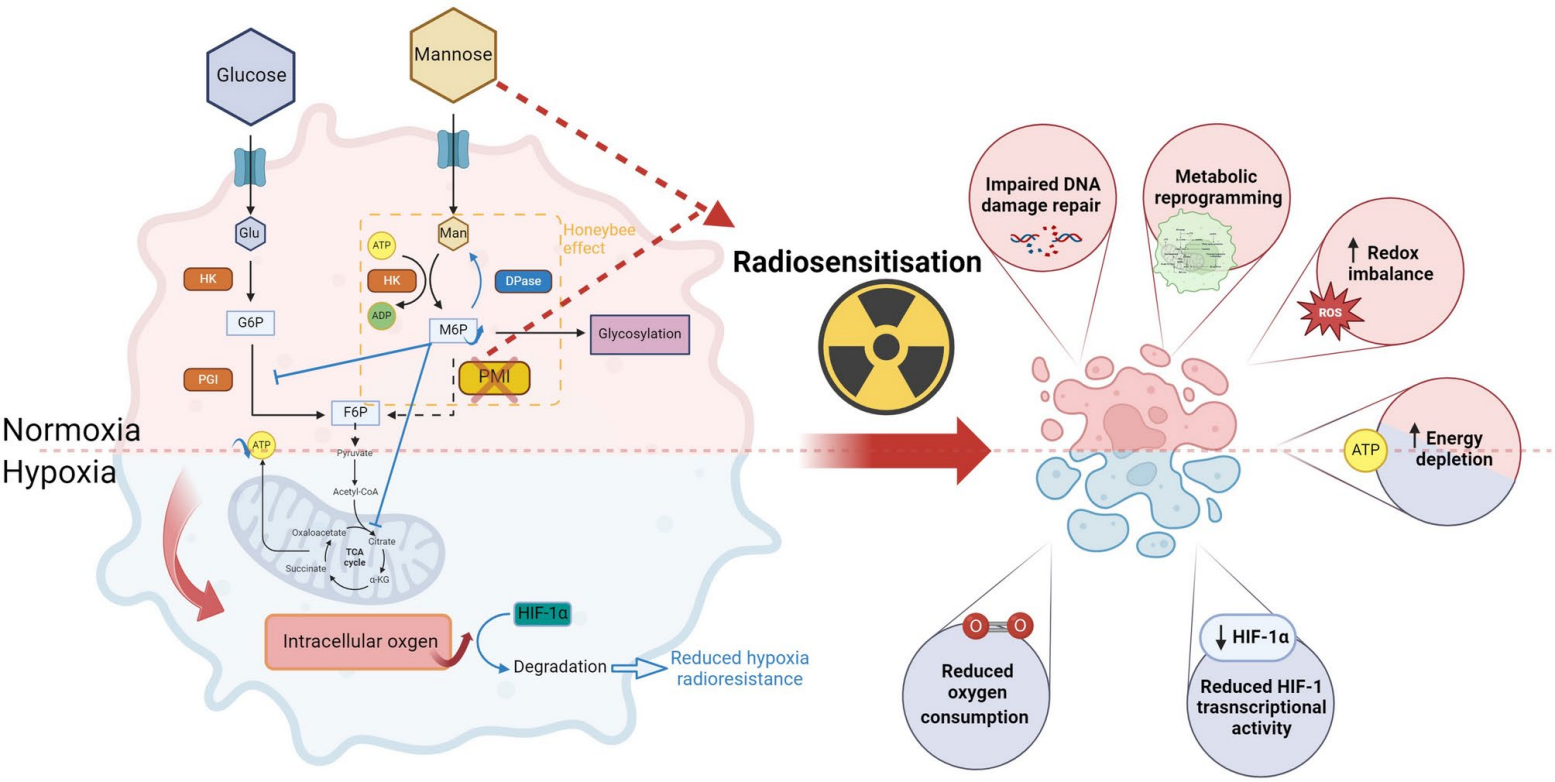
Mechanistic overview. Tumour cells exhibit monosaccharide addiction, providing a means for preferential and competitive uptake of mannose via glucose transporters (GLUT1/2). This figure outlines how altered mannose metabolism through PMI depletion enhances tumour cell radiation sensitivity. Mannose potentiates glycolytic disruption, particularly in PMI-depleted cells, driving metabolic reprogramming characterised by ATP depletion, redox imbalance, and impaired DNA damage repair. Under hypoxia, mannose and PMI ablation reduced oxygen consumption rate and supressed TCA cycle activity, effects that led to destabilised HIF-1α under hypoxic conditions. These metabolic driven adaptions ultimately helped counteract hypoxia-mediated radiation resistance, though elevated intracellular oxygen. Together, these mechanisms act together to sensitise various in vitro tumour models to radiation, responses that require further in vivo validation, positioning PMI as a promising target to improve radiotherapy outcomes. These findings provide compelling evidence for the continued clinical exploration of mannose as a radiosensitiser, particularly in the context of PMI ablation

This study highlights the potential of mannose as a metabolic radiosensitiser of HPV-negative HNSCC. While mannose alone shows modest anti-tumour effects, therapeutic impact is significantly amplified by PMI depletion. Ablation of this protein in the presence of mannose induces metabolic reprogramming, glycolytic suppression, inhibition of oxidative phosphorylation, and disruption of one-carbon metabolism. These changes impair DNA repair, increase residual DNA damage, sensitising tumours to radiation. A schematic mechanistic summary is provided in Fig. 7 illustrating the underpinning mechanisms contributing to mannose mediated radiosensitisation.

Our data clearly indicate that to capitalise on the full potential of mannose, supressing PMI function will be essential to overcome issues associated with unfavourable ADME characteristics, including rapid excretion of the unmetabolised sugar, resulting in a short plasma half-live (30 min–2 h). Given that HNSCC radiotherapy is delivered in multiple fractions over 6–7 weeks, intelligent strategies to exploit the full beneficial properties of mannose are required. This approach may negate the requirement for high-dose/frequent oral administration, which may trigger undesirable systemic side-effects, helping to fulfil the anti-tumour potential of a simple hexose sugar.

## Electronic supplementary material

Below is the link to the electronic supplementary material.

Supplementary Material 1

Supplementary Material 2

## Data Availability

No datasets were generated or analysed during the current study.
