# Supplementary material for: Mannose and PMI depletion overcomes radiation resistance in HPV-negative head and neck cancer
Source: Cell Commun Signal. 2025 Apr 21;23:189. doi: 10.1186/s12964-025-02204-0 (PMC12013184; doi:10.1186/s12964-025-02204-0)
Supplement: Supplementary file 1 — Supplementary Material 1 [file 12964_2025_2204_MOESM1_ESM.docx]

**Supplementary (S) Figures.**


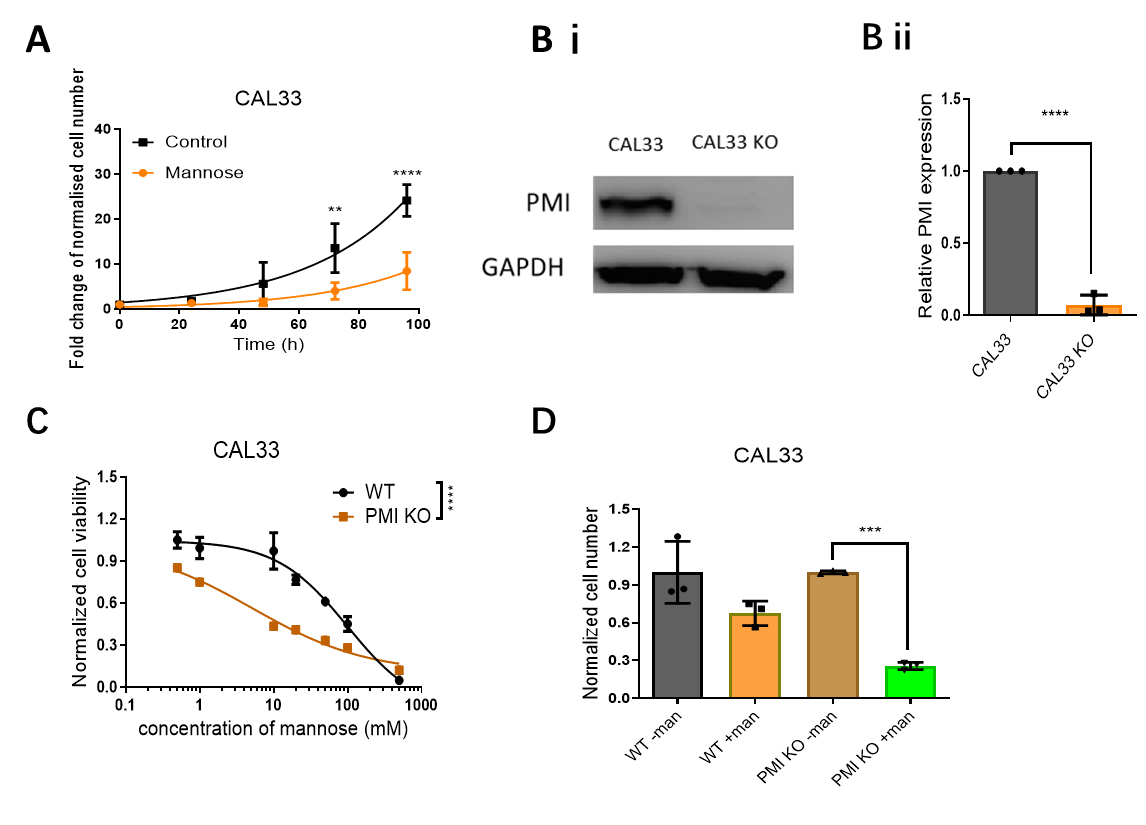


**Figure S1. PMI depletion enhances the anti-proliferative effect of mannose on CAL33 cells.**

**A)** Growth curve analysis of CAL33 cells +/- 20 mM mannose over 96 h. **B i)** Representative western blot image of CAL33 WT and KO cells. **B ii)** Densitometry analysis of PMI KO in CAL 33 cells. **C)** Concentration dependent (0 mM – 500 mM) impact of mannose on cell viability in PMI WT and KO CAL33 cells, measured 48 h post-treatment using Alamar blue reagent. **D)** Trypan blue exclusion assay demonstrating increased sensitivity to mannose (20 mM) following PMI KO over 48 h. Data information: In (A-D), data are presented as mean ± SD of three independent biological replicates. *p≤0.05 (two-way ANOVA – Bonferroni’s multiple comparisons test for **A**, unpaired t-test for **B** and one-way ANOVA – Tukey’s multiple comparisons test for **D**.


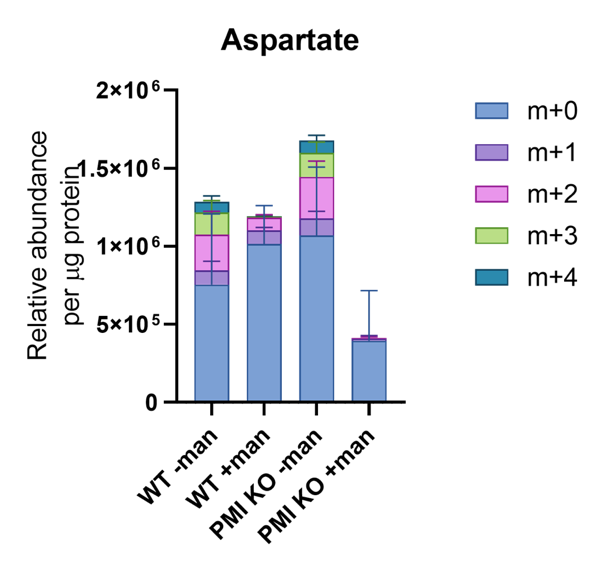
**Figure S2. Mannose inhibits intracellular aspartate abundance in PMI-depleted cells.** Quantitative analysis of the relative abundance of aspartate in uniformly labelled ^13^C_6_-Glc treated cells +/- mannose in WT and PMI KO FaDu cells treated. (n= 4 or 5 per group). Data are presented as mean ± SD.

**
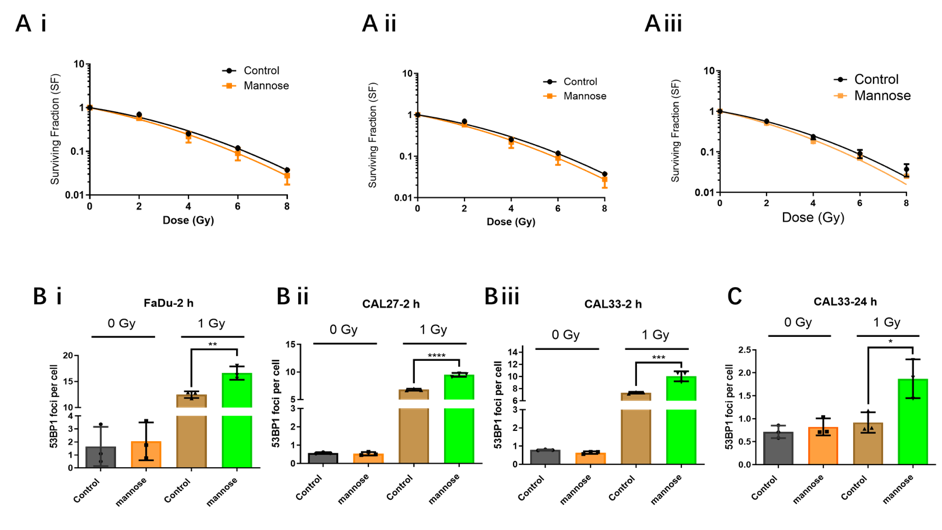
Figure S3.** **Radiation response of WT HNSCC CAL33 cells to radiation.** **A)** Clonogenic assay for CAL33 cells **i)** pre-treated, **ii)** post-treated or **iii)** exposed to prolonged mannose treatment. **B)** Quantification of 53BP1 double strand break foci in **i)** FaDu **ii)** CAL27 and **iii)** CAL33 cells 2 h post radiation (1 Gy) treatment. **C)** . Quantification of 53BP1 double strand break foci in CAL33 cells 24 h post radiation (1 Gy) treatment. Data information: A-C are presented as mean ± SD of three independent biological replicates. *p≤0.05 (unpaired Student t-test)


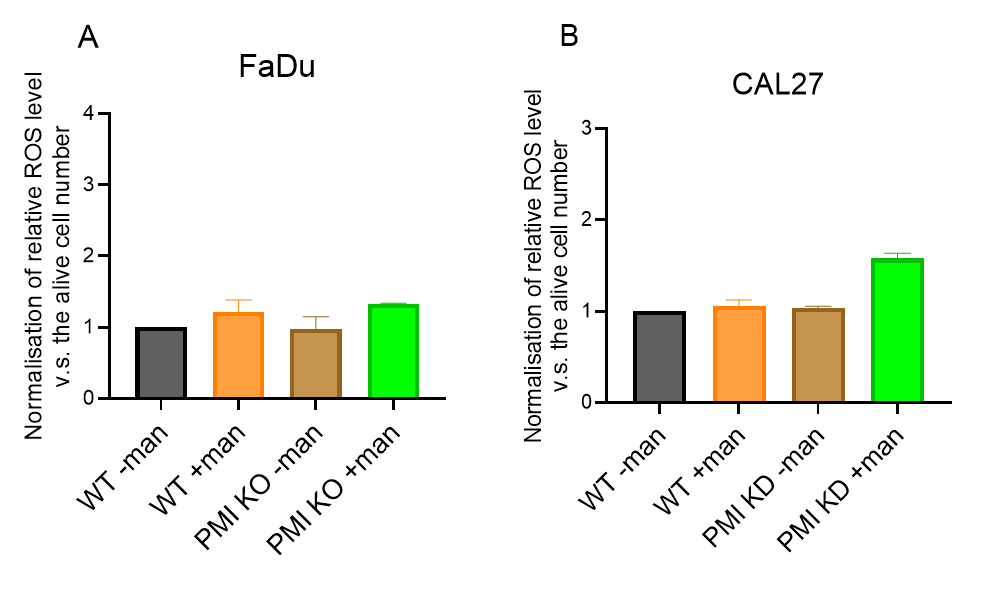
**Figure S4. Basal ROS level in unirradiated FaDu and CAL27 cells treated with mannose** +/- **PMI KO/KD.** Relative ROS levels detected using 2',7'-dichlorodihydrofluorescein diacetate (DCFH-DA) in WT and PMI KO/KD **A)** FaDu and **B)** CAL27 cells +/- mannose (20 mM) pre-treatment (24 h) without radiation. ROS levels were measured as control group. Data represent mean ± SD of three independent biological replicates.


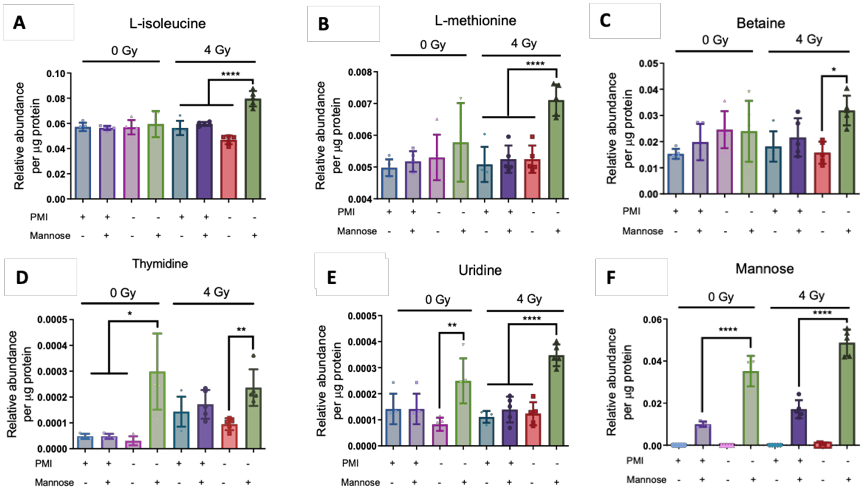


**Figure S5**. **Mannose combined with radiation causes the specific upregulation of several metabolites in PMI knockout cells.** Unlabelled LC-MS analysis of the metabolic profiles of WT and PMI KO FaDu cells treated with mannose, with or without radiation (4 Gy). **A-F)** Quantitative analysis of the relative abundance of key mannose/PMI KO/IR altered intracellular metabolites, including **A)** L-isoleucine; **B)** L-methionine; **C)** betaine; **D)** thymidine; **E)** uridine; and **F)** mannose (n= 5 per group). Data information: In (A-F), data are presented as mean ± SD. *p≤0.05 (one-way ANOVA – Tukey’s multiple comparisons test).


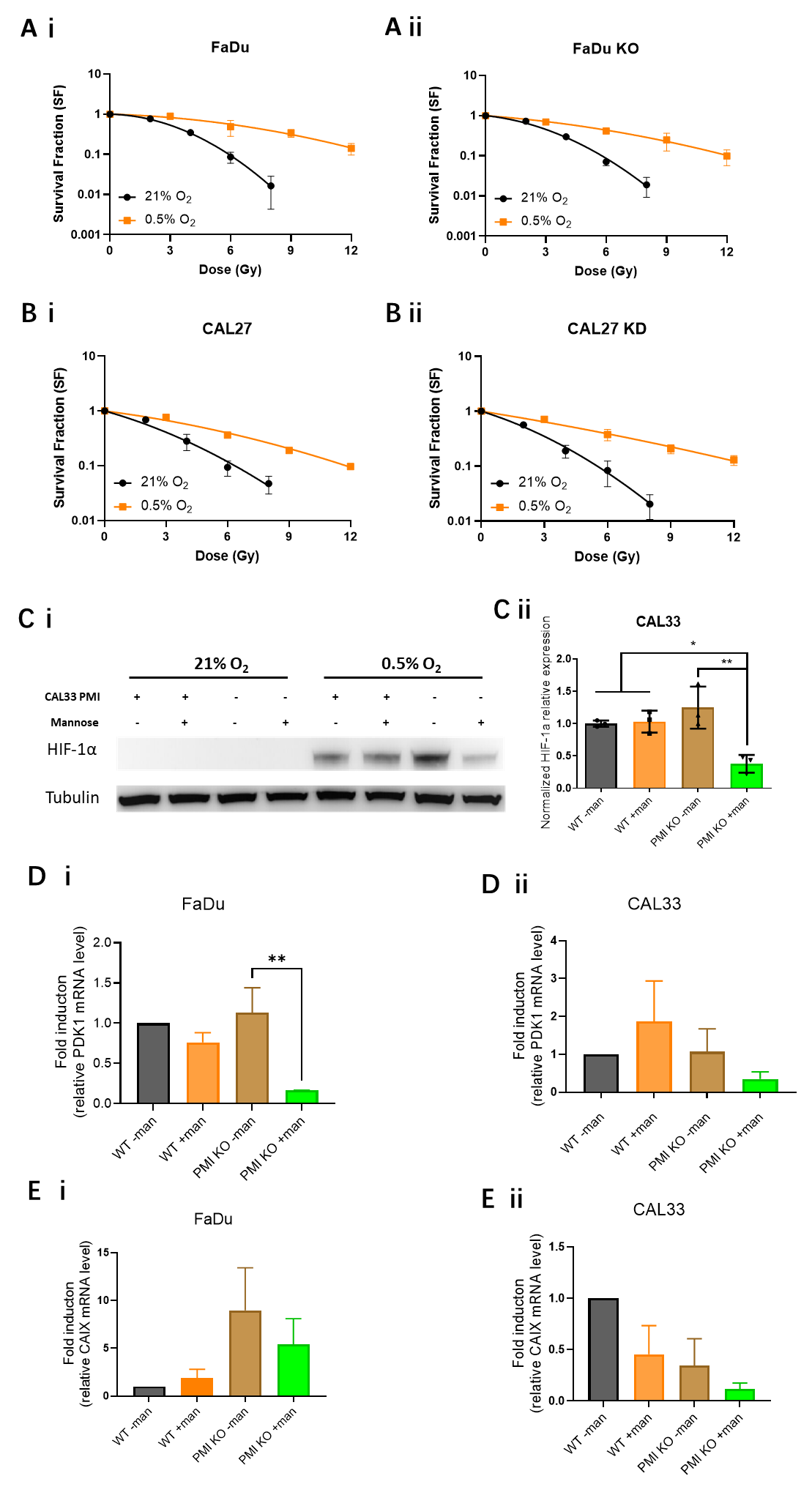


**Figure S6. Overcoming hypoxia-Induced radioresistance through HIF-1α suppression by mannose and PMI KO. A)** Clonogenic survival assay results for **i)** FaDu WT; **ii)**, FaDu KO; **B i)** CAL27 and **B ii)** CAL27 KD cells pretreated under either normoxia (21% O_2_) or hypoxic (0.5% O_2_) conditions for 4 h. **C i)** Representative western blot and **C ii)** densitometry analysis of HIF-1α levels in WT and PMI KO CAL33 cells +/- mannose for 24 h, followed by hypoxia (0.5% O_2_) exposure for 4 h. **D)** Relative mRNA expression levels of PDK-1 in WT and PMI KO/KD: **D i)** FaDu and **D ii)** CAL33 cells. **E)** Relative mRNA expression levels of CAIX in WT and PMI KO/KD: **E i)** FaDu and **E ii)** CAL33 cells. mRNA expression levels were normalised against RPL13A, used as a housekeeping gene, and presented as fold change relative to the control group. Samples were collected under hypoxic conditions (0.5% O_2_ for 4 h). Data information: Data in A-C is presented as mean ± SD of three independent biological replicates. *p≤0.05 - one-way ANOVA, with Tukey’s multiple comparisons test.

**
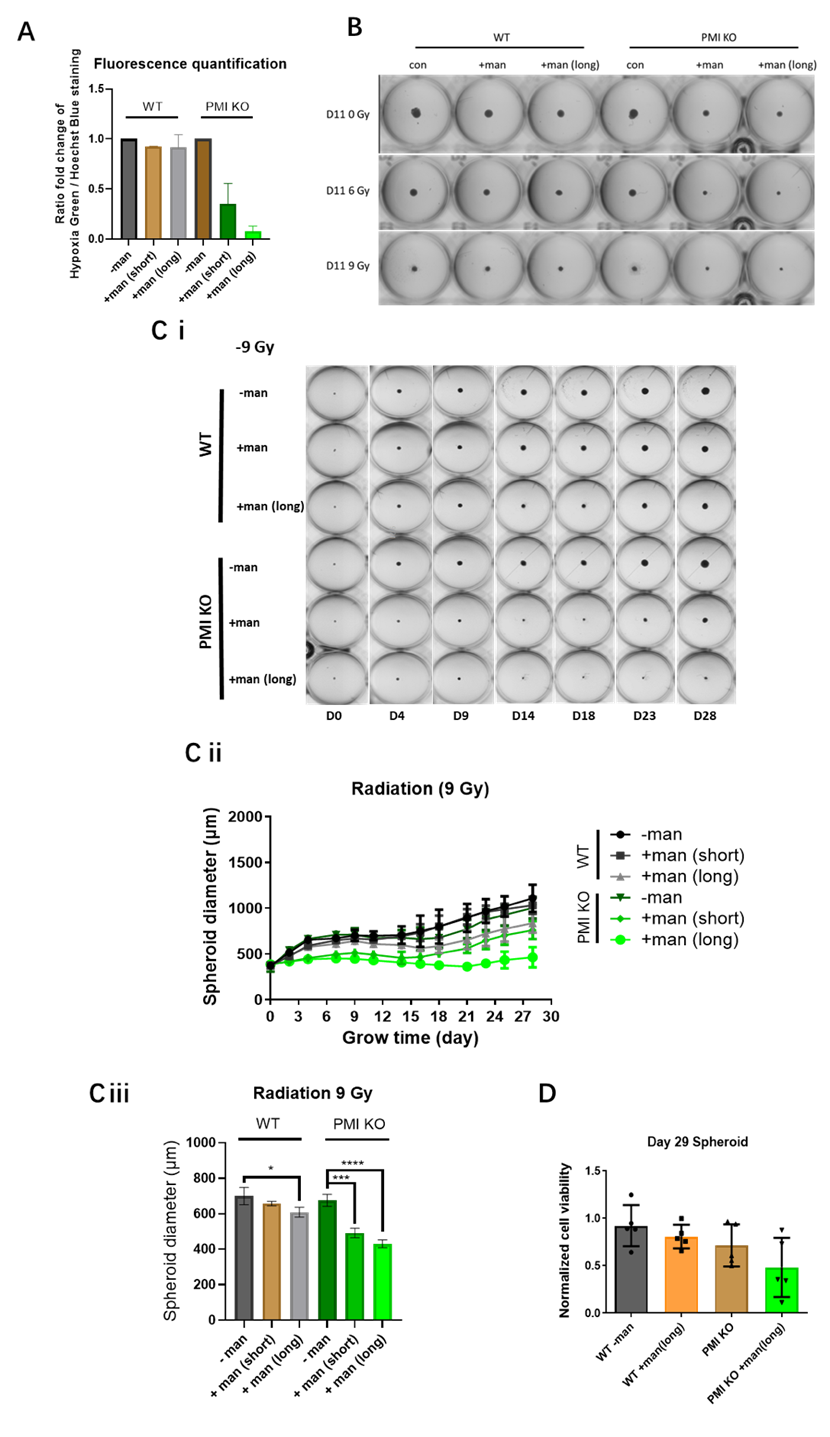
Figure S7. Mannose sensitises PMI KO HNSCC tumourspheres to radiation.** **A)** Size-matched (~600 μm) FaDu (WT/KO) tumourspheres pre-treated with 20 mM mannose. Ratio fold change of Hypoxia Green / Hoechst Blue staining, with tumoursphere fluorescence quantified using ImageJ (n=2 per group). **B)** Representative images of FaDu parental and PMI KO tumoursphere treated with short term mannose (48 h) and long-term mannose (sustained) with 0 Gy, 6 Gy and 9 Gy radiation in Day 11.**C i&ii)** Images and diameters of 9 Gy irradiated FaDu and FaDu KO tumourspheres collected three times weekly until 28 days. **C iii)** Differential tumoursphere diameter on Day 11 for WT and PMI KO tumourspheres treated with mannose for short-term (48 h) or long-term (sustained) exposure following 9 Gy radiation treatment. **D)** tumourspheres viability on day 28, assessed using Alamar Blue reagent to evaluate cell viability. Data information: Data in C & D are presented as mean ± SD of three independent biological replicates. *p≤0.05 (one-way ANOVA – Tukey’s multiple comparisons test)**.**

**Table S1.** Respective dose in Gy required to kill 90% of cells under either normoxia (21% O_2_) or hypoxia (0.5% O_2_). Oxygen enhancement factors (OER) were calculated at a surviving fraction of 0.1 between normoxic and hypoxic irradiated cells.

| SF=0.1 | Dose (hypoxia)/Gy | Dose (normoxia)/Gy | OER |
| --- | --- | --- | --- |
| FaDu | 12.83 | 5.87 | 2.19 |
| FaDu KO | 12.08 | 5.79 | 2.09 |
| CAL27 | 11.77 | 6.35 | 1.85 |
| CAL27 KD | 13.02 | 5.51 | 2.36 |
