# Supplementary figures and images for: Mannose and PMI depletion overcomes radiation resistance in HPV-negative head and neck cancer
Source: Cell Commun Signal. 2025 Apr 21;23:189. doi: 10.1186/s12964-025-02204-0 (PMC12013184; doi:10.1186/s12964-025-02204-0)

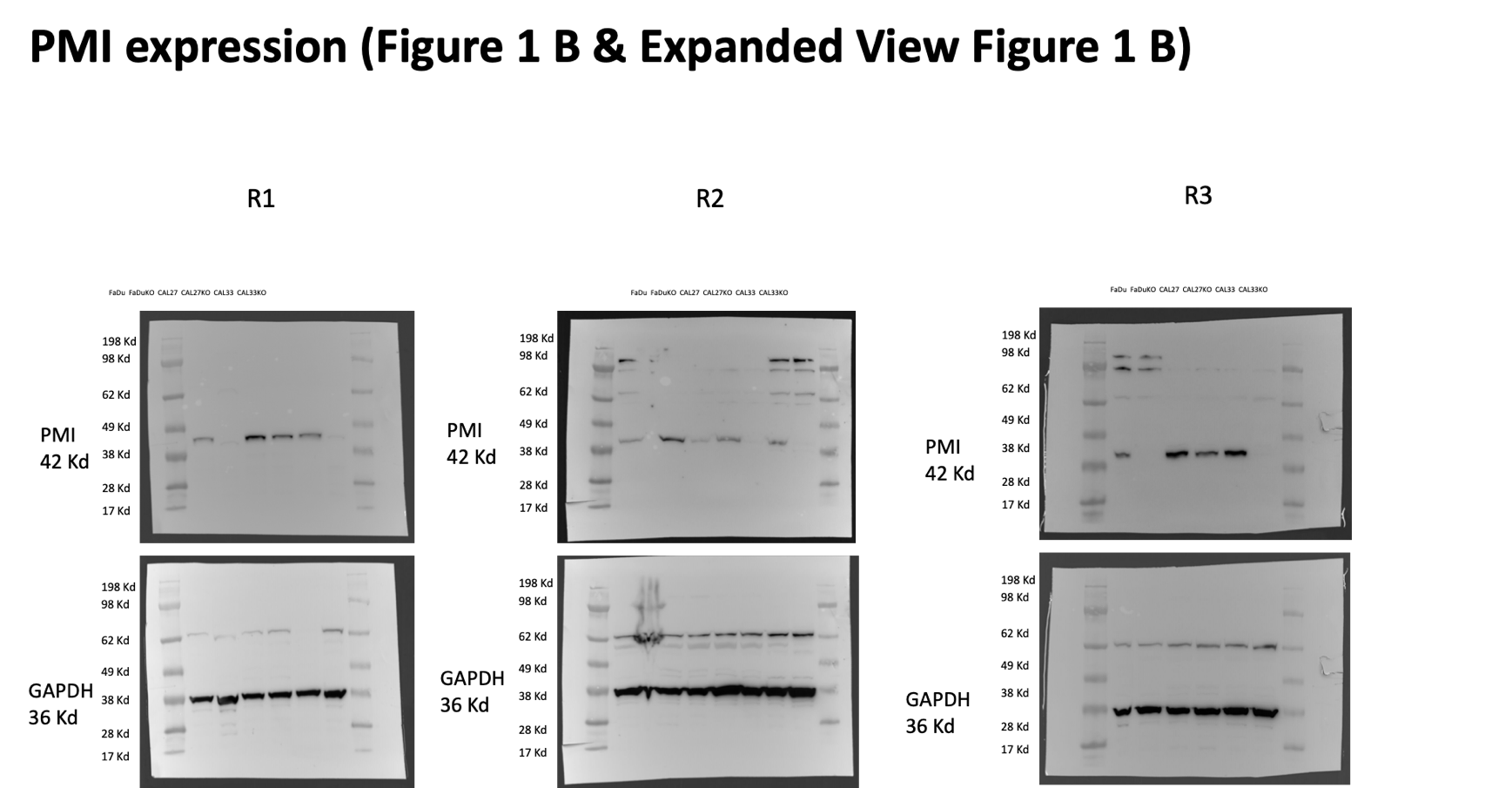


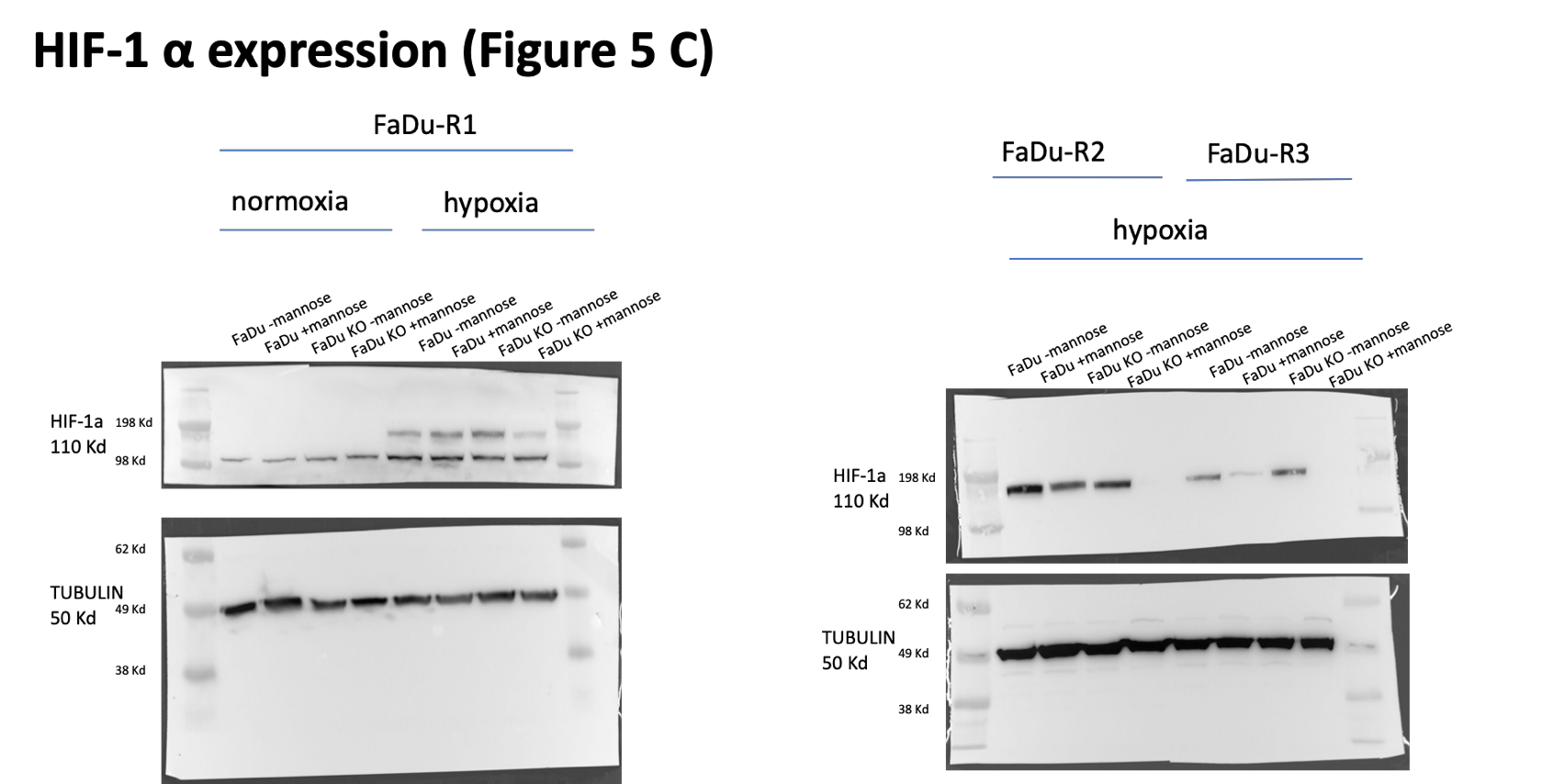


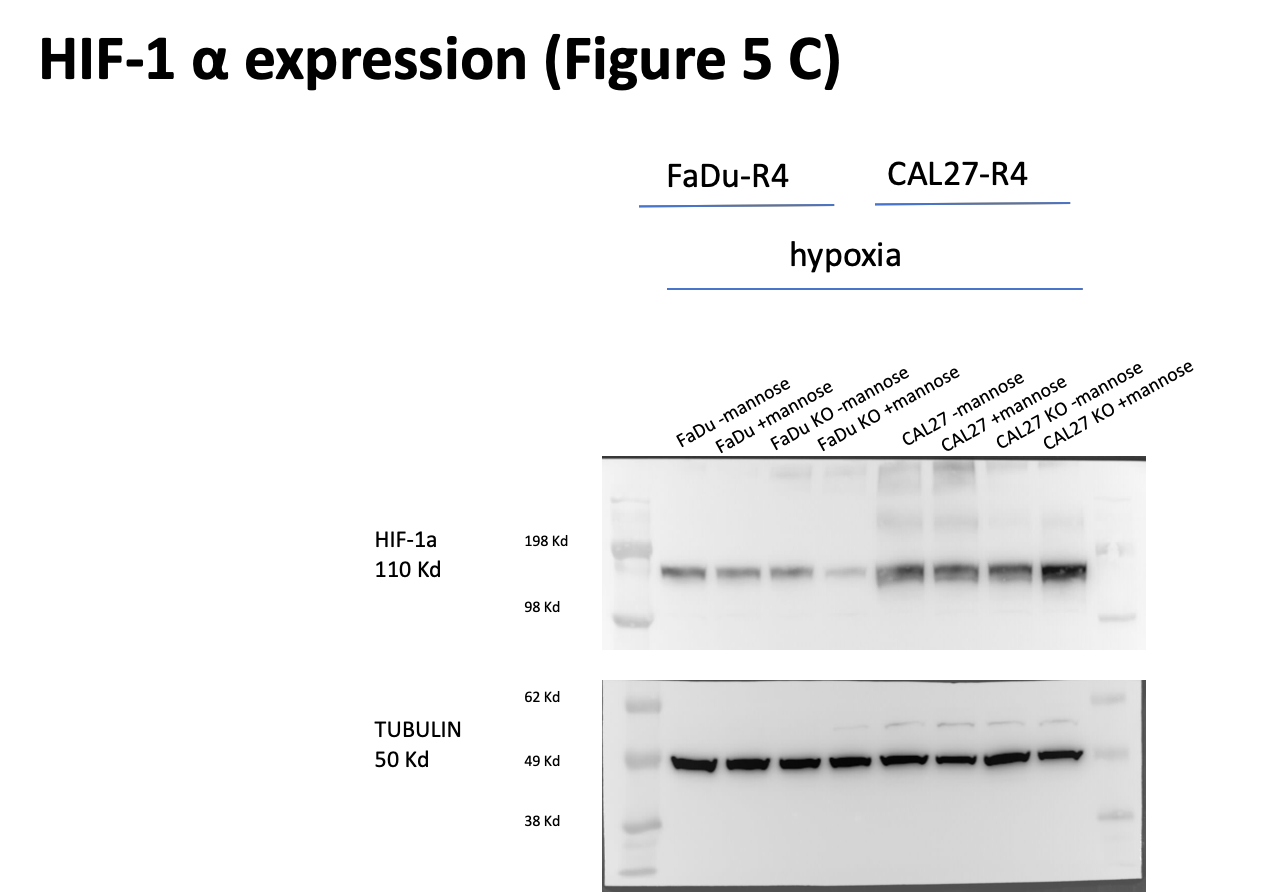


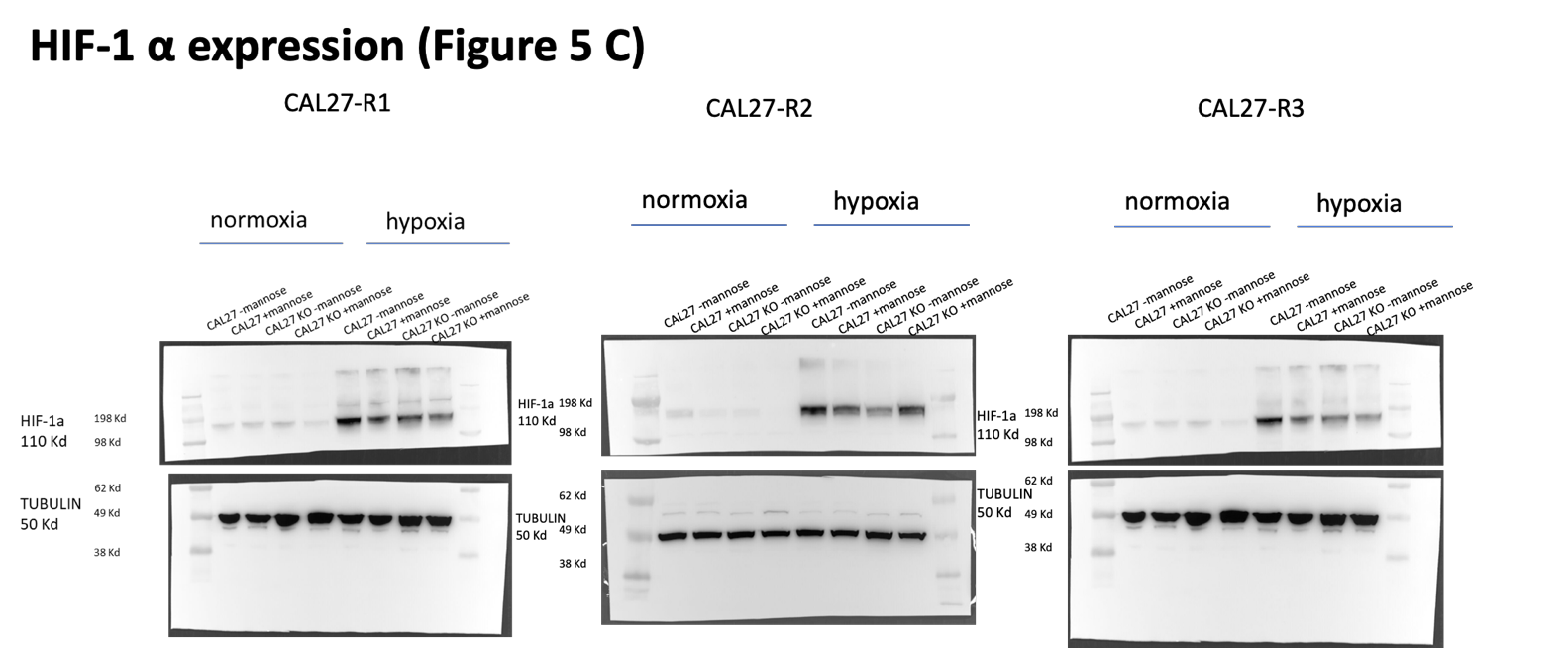


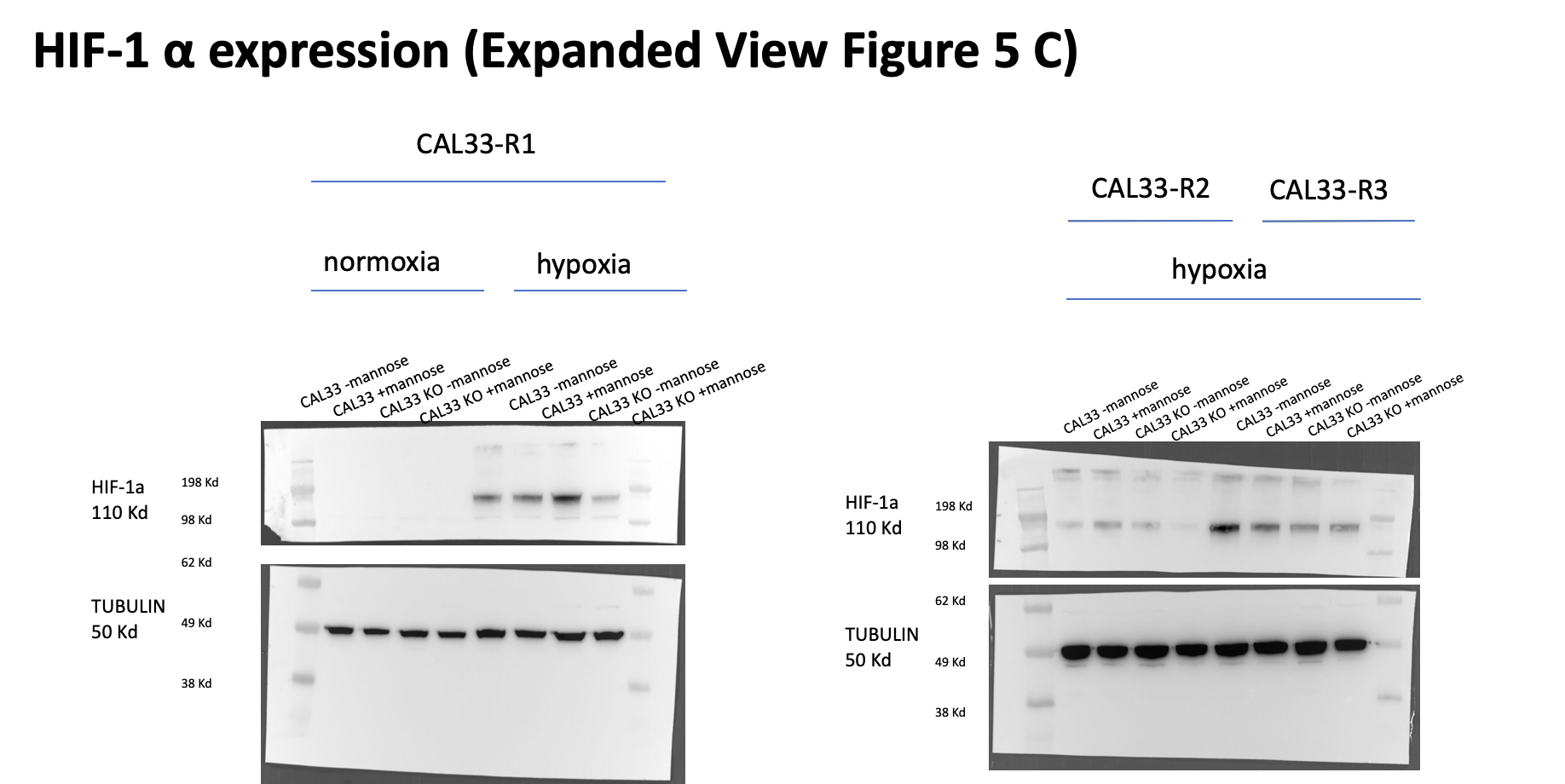

Supplement: Supplementary file 2 — Supplementary Material 2 [file 12964_2025_2204_MOESM2_ESM.docx]
